# Isolation, Purification and Tyrosinase Inhibitory Activity of Anthocyanins and Their Novel Degradation Compounds from *Solanum tuberosum* L.

**DOI:** 10.3390/molecules29071492

**Published:** 2024-03-27

**Authors:** Jian Ouyang, Na Hu, Honglun Wang

**Affiliations:** 1 Qinghai Provincial Key Laboratory of Tibetan Medicine Research and CAS Key Laboratory of Tibetan Medicine Research, Northwest Institute of Plateau Biology, Xining 810008, China; ygzjj@126.com (J.O.); huna@nwipb.cas.cn (N.H.); 2 Huzhou China-Science Innovation Centre of Plateau Biology, Huzhou 313000, China; 3 University of Chinese Academy of Sciences, Beijing 100049, China

**Keywords:** acylated anthocyanins, norpetanin, 4-*O*-(*p*-coumaryl) rhamnose, molecular docking, petanin, tyrosinase inhibitor

## Abstract

To explore the composition of anthocyanins and expand their biological activities, anthocyanins were systematically isolated and purified from tubers of *Solanum tuberosum* L., and their tyrosinase inhibitory activity was investigated. In this study, two new anthocyanin degradation compounds, norpetanin (**9**) and 4-*O*-(*p*-coumaryl) rhamnose (**10**), along with 17 known anthocyanins and their derivatives, were isolated and purified from an acid-ethanolic extract of fresh purple potato tubers. Their structures were elucidated via 1D and 2D NMR and HR-ESI-MS and compared with those reported in the literature. The extracts were evaluated for anthocyanins and their derivatives using a tyrosinase inhibitor screening kit and molecular docking technology, and the results showed that petanin, norpetanin, 4-*O*-(*p*-coumaryl) rhamnose, and lyciruthephenylpropanoid D/E possessed tyrosinase inhibitory activity, with 50% inhibiting concentration (IC_50_) values of 122.37 ± 8.03, 115.53 ± 7.51, 335.03 ± 12.99, and 156.27 ± 11.22 μM (Mean ± SEM, *n* = 3), respectively. Furthermore, petanin was validated against melanogenesis in zebrafish; it was found that it could significantly inhibit melanin pigmentation (*p* < 0.001), and the inhibition rate of melanin was 17% compared with the normal group. This finding may provide potential treatments for diseases with abnormal melanin production, and high-quality raw materials for whitening cosmetics.

## 1. Introduction

The potato (*Solanum tuberosum* L.) is the fourth most important staple crop worldwide and originated from the highlands of the equatorial Andes in South America [1,2]. The potato is a diverse crop that includes varieties with white, yellow, and colored flesh (red, blue, and purple). These differences in flesh color result from significant differences in the phytochemical composition of crops [3,4]. Yellow-flesh potatoes are characterized by high levels of carotenoids, whereas red-, blue-, and purple-flesh potatoes contain large amounts of anthocyanins [5]. Therefore, potatoes rich in pigments are a good source of anthocyanins [6].

Colored potatoes are a rich source of anthocyanins, especially the acylated derivatives, which account for more than 98% of the total anthocyanins [7]. The red potato contains mainly the acylated side of pelargonidin, while the purple potato contains mainly the acylated side of petunidin and peonidin, whereas delphinidin and malvidin have fewer acylated glycosides. Red tubers contain mostly pelargonidin 3-*O*-(*p*-coumaroyl-rutinoside)-5-*O*-glucoside and lesser amounts of peonidin 3-*O*-(*p*-coumaroyl-rutinoside)-5-*O*-glucoside. Light to medium purple tubers contain petunidin 3-*O*-(*p*-coumaroyl-rutinoside)-5-*O*-glucoside (1.00~2.00 mg g^−1^, FW) and small amounts of malvidin 3-*O*-(*p*-coumaroyl-rutinoside)-5-*O*-glucoside, while dark purple–black tubers contained similar levels of petunidin 3-*O*-(*p*-coumaroyl-rutinoside)-5-*O*-glucoside, together with much higher concentrations of malvidin-3-*O*-(*p*-coumaroyl-rutinoside)-5-*O*-glucoside [8]. Caffeoyl anthocyanins were first identified in Norwegian potatoes, containing petunidin 3-*O*-[6-*O*-(4-*O*-*E*-caffeoyl-*O*-α-rhamnopyranosyl)-*β*-glucopyranoside]-5-*O*-*β*-glucopyranoside (10%), peonidin 3-*O*-[6-*O*-(4-*O*-*E*-caffeoy1-*O*-α-rhamnopyranosyl)-β-glucopyranoside]-5-*O*-β-glucopyranoside (6%), petunidin 3-*O*-[6-*O*-(4-*O*-*E*-*p*-coumaroyl-*O*-α-rhamnopyranosyl)-*β*-glucopyranoside]-5-*O*-*β*-g1ucopyranoside (petanin, 37%), and peonidin 3-*O*-[6-*O*-(4-*O*-*E*-*p*-coumaroyl-*O*-α-rhamnopyranosyl)-*β*-glucopyranoside]-5-*O*-*β*-glucopyranoside (25%) [9]. The study of polyphenol composition in colored potato skins found that the total anthocyanin content in purple potatoes was 0.863 ± 0.005–1.39 ± 0.01 mg g^−1^ (dry weight, DW), including acylated pelargonidin, peonidin, malvidin, and petunidin anthocyanins, as shown in Table 1 [7,10,11,12]. Thus, the types of anthocyanins contained in a crop are closely related to the variety of potato, and the content varies greatly among different varieties and regions.

Anthocyanins are a type of flavonoid and glycosidic water-soluble pigment that not only impart red, purple, and blue colors to many fruits, flowers, and tubers, but also confer physiological benefits, reflected in their antioxidant [13,14,15], anti-inflammatory [16,17], hypoglycaemic [18,19], and whitening properties [20,21,22]. In particular, tyrosinase inhibitors are gaining research interest as melanin pigmentation can be blocked by inhibiting tyrosinase, a rate-limiting enzyme in melanin production [23,24]. Extracts from the seed coat of black soya beans have been reported to possess anti-human tyrosinase activity, and a good correlation has been found between such anti-human tyrosinase activity and the cyanidin 3-*O*-glucoside content of coat extracts [25]. Radio frequency-assisted enzymatic extraction of anthocyanins from *Akebia trifoliata* (Thunb.) Koidz. flowers showed tyrosinase inhibitory activity (14.67 kojic acid equivalents/g extract) [26]. The IC_50_ value of anthocyanins from red rice bran, in which tyrosinase inhibitory activity was observed, was reported to be 4.26 μg mL^−1^ [27]. Anthocyanins purified from *Lycium ruthenicum* Murr. had inhibitory effects on tyrosinase monophenolase (IC_50_ = 1.483 ± 0.058 mg mL^−1^), and the type of inhibition was competitive (K_i_ = 39.83 ± 1.4 mg mL^−1^) [28]. Moreover, petunidin 3-*O*-glucoside may act as a tyrosinase inhibitor to block melanin production, and has inhibitory ratios exceeding 55% of the control value at 50 µM, showing dose-dependent inhibitory activity with an IC_50_ value of 10.3 ± 1.0 µM [29]. Additionally, anthocyanins from *Hibiscus syriacus* L. inhibit melanogenesis by activating the extracellular regulated protein kinases signaling pathway [30]. Therefore, anthocyanins have good potential for tyrosinase inhibitory activity, and thus anti-melanogenesis.

In this study, anthocyanin components from *Solanum tuberosum* L. were isolated, purified, and prepared using chromatography, spectroscopy, and NMR techniques. Then, their tyrosinase inhibitory activities were evaluated using tyrosinase inhibitor screening kits, molecular docking, dynamic simulation, and through examination of their toxicity and anti-melanogenic effects in zebrafish. These results are expected to provide a more comprehensive understanding of the composition of anthocyanins in purple potato, their tyrosinase inhibitory activity, and potential for anti-melanogenic effects, and promote the wider use of purple potato in these contexts.

## 2. Results and Discussion

### 2.1. Compounds Structure Identification

**Figure 1:**
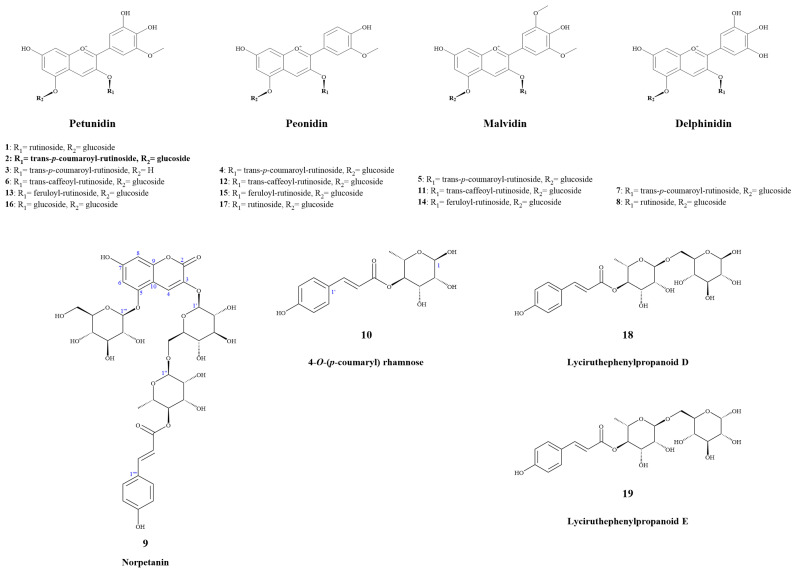
Anthocyanins and their novel degradation compounds from *Solanum tuberosum* L.

Anthocyanins and their novel degradation compounds (**9** and **10**) were extracted from fresh slices of potato tubers using acidic water-ethanol, enriched with macroporous resin, and purified via semi-preparative chromatography, as shown in Figure 1.

#### 2.1.1. Resolution and Identification of New Compounds

**Figure 2:**
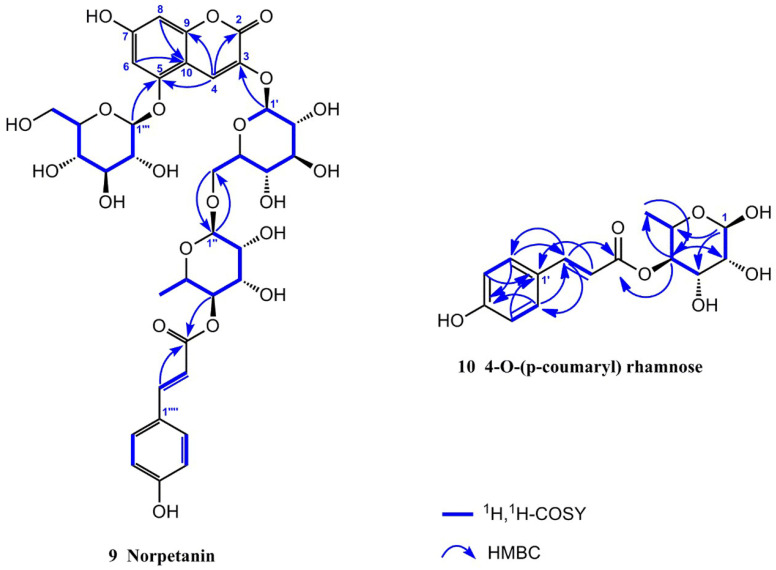
Structures, key HMBC and ^1^H, ^1^H-COSY correlation of compounds **9** and **10**.

Compound **9** was obtained as a yellowish amorphous powder with a molecular formula of C_36_H_42_O_21_, as determined using HR-ESI-MS (*m*/*z* 833.2117 [M + Na]^+^, calcd. 833.2111). It had 16 degrees of unsaturation. The ^1^H NMR spectrum (Table 2) in CD_3_OD/CF_3_COOD (9:1) of **9** exhibited a set of *trans*-*p*-coumaroyl signals at *δ*_H_ 7.48 (2H, d, *J* = 8.4 Hz, H-2′′′′/H-6′′′′), 6.80 (2H, d, *J* = 8.4 Hz, H-3′′′′/H-5′′′′), 7.60 (1H, d, *J* = 15.9 Hz, H-7′′′′) and 6.34 (1H, d, *J* = 15.9 Hz, H-8′′′′), one aromatic or olefinic proton singlet at *δ*_H_ 7.69 (1H, s, H-4), a set of 1,3,4,5-tetrasubstituted benzene ring signals at *δ*_H_ 6.60 (1H, d, *J* = 1.4 Hz, H-6) and 6.37 (1H, d, *J* = 1.4 Hz, H-8), two β-glucopyranosyl anomeric protons at *δ*_H_ 5.07 (1H, d, *J* = 7.0 Hz, H-1′) and 4.95 (1H, d, *J* = 7.8 Hz, H-1′′′), an α-rhamnopyranosyl anomeric proton at *δ*_H_ 4.76 (1H, br s, H-1′′), an acylated methine triplet at *δ*_H_ 4.94 (1H, t-like, *J* = 9.5 Hz, H-4′′), as well as a rhamnosyl methyl doublet in the up-field region. The ^13^C NMR spectrum (Table 2) showed 36 carbon resonances, including a set of *trans*-*p*-coumaroyl carbons and 18 carbon signals assignable to three sugar moieties, whereas the remaining nine unsolved resonances originated from the aglycone core. The above NMR features were generally similar to those of petanin [31]; the main difference was that the ring B signals in the anthocyanin core were absent in **9**, based on which the structure was inferred. It was inferred that the ring B moiety was oxidized and degraded to derive an unusual 3,5,7-trioxygen-substituted coumarin core. This inference was confirmed by the HMBC correlations (Figure 2) from H-4 to C-2 [*δ*_C_ 160.4 (s)], C-5 [*δ*_C_ 155.4 (s)], and C-9 [*δ*_C_ 153.3 (s)], H-6 and H-8 to C-10 [*δ*_C_ 104.4 (s)], and from H-1′ to C-3 [*δ*_C_ 139.0 (s)]. The resulting structure was further verified via careful analysis of the ^1^H NMR, ^13^C NMR, HR-ESI-MS, HSQC, HMBC, and ^1^H,^1^H-COSY correlations shown in Figure 2 and Figures S1–S6. Therefore, the structure of compound **9** was established, as shown in Figure 1, and the compound was named **norpetanin**. To the best of our knowledge, compound **9** represents a rare class of ring B anthocyanins.

Compound **10** was obtained as a yellowish amorphous powder with a molecular formula of C_15_H_18_O_7_ and seven degrees of unsaturation. The ^1^H and ^13^C NMR spectrum (Table 3) in CD_3_OD of **10** exhibited a set of *trans*-*p*-coumaroyl signals at *δ*_H_ 7.47 (2H, d, *J* = 8.5 Hz, H-2′/H-6′), 6.80 (2H, d, *J* = 8.5 Hz, H-3′/H-5′), 7.64 (1H, d, *J* = 15.9 Hz, H-7′), and 6.36 (1H, d, *J* = 15.9 Hz, H-8′), an α-rhamnopyranosyl anomeric proton at *δ*_H_ 5.04 (1H, br s, H-1), an acylated methine triplet at *δ*_H_ 5.02 (1H, t-like, *J* = 9.7 Hz, H-4), and a rhamnosyl methyl doublet in the up-field region. The inference was confirmed via the HMBC correlations from H-1 to C-3 [*δ*_C_ 70.3 (d)], C-5 [*δ*_C_ 67.3 (d)], from H-2 to C-3 and C-4 [*δ*_C_ 75.7 (d)], from H-3 to C-2 [*δ*_C_ 73.1 (d)], from H-4 to C-3, C-5, C-6 [*δ*_C_ 18.0 (q)], and C-9′ [*δ*_C_ 169.0 (s)], from H-6 to C-4 and C-5, from H-3′ and H-5′ to C-1′ [*δ*_C_ 127.2 (s)] and C-4′ [*δ*_C_ 161.3 (s)], from H-2′ and H-6′ to C-4′ and C-7′ [*δ*_C_ 146.8 (d)], from H-7′ to C-2′, 6′ [*δ*_C_ 131.2 (d)], C-8′ [*δ*_C_ 115.2 (d)], and C-9′ [*δ*_C_ 169.0 (s)], from H-8′ to C-1′ and C-9′. The resulting structure was further verified through careful analysis of the HSQC, HMBC, and ^1^H,^1^H-COSY correlations shown in Figure 2 and Figures S7–S11. Therefore, the structure of compound **10** was established (Figure 1) and it was named **4-*O*-(*p*-coumaryl) rhamnose**.

#### 2.1.2. First Report of NMR Data for Known Compounds

Compound **3**, which was obtained as an amorphous purple powder, possessed the molecular formula C_37_H_39_O_18_^+^ and had 19 degrees of unsaturation. The ^1^H-NMR spectrum (CD_3_OD:CF_3_COOD ≈ 9:1, 600 MHz) was as follows: *δ*_H_ 8.92 (1H, s, H-4), 7.93 (1H, d, *J* = 1.6 Hz, H-2′), 7.77 (1H, d, *J* = 1.6 Hz, H-6′), 7.56 (1H, d, *J* = 15.9 Hz, H-7′′′′), 7.38 (2H, d, *J* = 8.5 Hz, H-2′′′′, H-6′′′′), 6.83 (1H, br s, H-8), 6.80 (2H, d, *J* = 8.5 Hz, H-3′′′′, H-5′′′′), 6.65 (1H, br s, H-6), 6.20 (1H, d, *J* = 15.9 Hz, H-8′′′′), 5.35 (1H, d, *J* = 7.6 Hz, H-1′′), 4.94 (1H, t, *J* = 9.4 Hz, H-4′′′), 4.71 (1H, br s, H-1′′′), 3.97 (3H, s, 3′-OCH_3_), 0.98 (3H, d, *J* = 6.2 Hz, H-6′′′), as shown in Figure S12. Through comparison with research data [32], compound **3** was identified as **petunidin 3-*O*-*trans*-*p*-coumaroylrutinoside**.

Compound **12**, obtained as a purple amorphous powder, possessed the molecular formula C_43_H_49_O_23_^+^ and 19 degrees of unsaturation. The ^1^H-NMR spectrum (CD_3_OD:CF_3_COOD ≈ 9:1, 600 MHz) was as follows: *δ*_H_ 8.99 (1H, s, H-4), 8.28 (1H, dd, *J* = 8.8, 1.9 Hz, H-6′), 8.20 (1H, d, *J* = 1.9 Hz, H-2′), 7.49 (1H, d, *J* = 15.9 Hz, H-7′′′′′), 7.01–7.09 (4H, overlapped, H-6, H-8, H-5′, H-2′′′′′), 6.89 (1H, br d, *J* = 8.2 Hz, H-6′′′′′), 6.77 (1H, d, *J* = 8.2 Hz, H-5′′′′′), 6.20 (1H, d, *J* = 15.9 Hz, H-8′′′′′), 5.50 (1H, d, *J* = 8.0 Hz, H-1′′), 5.19 (1H, d, *J* = 7.8 Hz, H-1′′′′), 4.90 (1H, t, *J* = 9.7 Hz, H-4′′′), 4.71 (1H, br s, H-1′′′), 4.00 (3H, s, 3′-OCH_3_), 0.99 (3H, d, *J* = 6.2 Hz, H-6′′′), as shown in Figure S13. It was named **peonidin 3-*O*-*trans*-caffeoylrutinoside-5-*O*-glucoside** [33].

Compound **15**, obtained as a purple amorphous powder, possessed the molecular formula C_44_H_51_O_23_^+^ and 20 degrees of unsaturation. The ^1^H-NMR spectrum (CD_3_OD:CF_3_COOD ≈ 9:1, 600 MHz) was as follows: *δ*_H_ 9.00 (1H, s, H-4), 8.29 (1H, dd, *J* = 8.8, 1.8 Hz, H-6′), 8.22 (1H, d, *J* = 1.8 Hz, H-2′), 7.56 (1H, d, *J* = 15.9 Hz, H-7′′′′′), 7.15 (1H, d, *J* = 1.6 Hz, H-2′′′′′), 7.01–7.10 (4H, overlapped, H-6, H-8, H-5′, H-6′′′′′), 6.80 (1H, d, *J* = 8.1 Hz, H-5′′′′′), 6.30 (1H, d, *J* = 15.9 Hz, H-8′′′′′), 5.47 (1H, d, *J* = 7.8 Hz, H-1′′), 5.18 (1H, d, *J* = 7.8 Hz, H-1′′′′), 4.90 (1H, t, *J* = 9.7 Hz, H-4′′′), 4.70 (1H, br s, H-1′′′), 4.00 (3H, s, 3′-OCH_3_), 3.87 (3H, s, 3′′′′′-OCH_3_), 0.98 (3H, d, *J* = 6.2 Hz, H-6′′′), as shown in Figure S14. Based on comparison and analysis of research data [33,34], **15** was identified as **peonidin 3-*O*-feruloylrutinoside-5-*O*-glucoside**.

#### 2.1.3. Identification of Known Compounds

Compounds **1**–**8** and **11**–**19** were identified as **petunidin 3-*O*-rutinoside-5-*O*-glucoside** (**1**) [35], **petanin** (**2**), **petunidin 3-*O*-trans-p-coumaroylrutinoside** (**3**) [32], **peonidin 3-*O*-trans-p-coumaroylrutinoside-5-*O*-glucoside** (**4**) [33], **malvidin 3-*O*-trans-p-coumaroylrutinoside-5-*O*-glucoside** (**5**) [35], **petunidin 3-*O*-trans-caffeoylrutinoside-5-*O*-glucoside** (**6**) [35], **delphinidin 3-*O*-trans-p-coumaroylrutinoside-5-*O*-glucoside** (**7**) [36], **delphinidin 3-*O*-rutinoside-5-*O*-glucoside** (**8**) [35], **malvidin 3-*O*-trans-caffeoylrutinoside-5-*O*-glucoside** (**11**) [37], **peonidin 3-*O*-trans-caffeoylrutinoside-5-*O*-glucoside** (**12**) [33], **petunidin 3-*O*-feruloylrutinoside-5-*O*-glucoside** (**13**) [34], **malvidin 3-*O*-feruloylrutinoside-5-*O*-glucoside** (**14**) [34], **peonidin 3-*O*-feruloylrutinoside-5-*O*-glucoside** (**15**) [33,34], **petunin** (**16**) [38], **peonidin 3-*O*-rutinoside-5-*O*-glucoside** (**17**) [33], **lyciruthephenylpropanoid D** (**18**) [39], and **lyciruthephenylpropanoid E** (**19**) [39] via NMR, spectroscopic, mass spectrometric, and chromatographic analyses and comparison with compounds reported in the literature, as shown in Figures S16 and S17 and Table S1.

The anthocyanins in purple potato were found to be mainly petunidin anthocyanins, such as petunidin 3-*O*-rutinoside-5-*O*-glucoside, petanin, petunidin 3-*O*-trans-*p*-coumaroylrutinoside, petunidin 3-*O*-trans-caffeoylrutinoside-5-*O*-glucoside, petunidin 3-*O*-feruloylrutinoside-5-*O*-glucoside, and petunin [9,10,11]. However, there were slight differences in these results as compared to previous studies. For instance, in previous studies, the main anthocyanins of purple clones (UACH 0917, Shetland Black, and Violetta) were found to be malvidin, petunidin, and cyanidin, pelargonidin 3-*O*-*p*-coumaroylrutinoside-5-*O*-glucoside (0~0.0387 mg g^−1^), and petunidin 3-*O*-glucoside has also been identified in purple potatoes (Heijingang) [10,12,40]. Nevertheless, these differences may be related to potato varieties, analysis and identification methods, and producing areas.

Although lyciruthephenylpropanoid D/E was first identified in *Lycium ruthenicum* Murr., lyciruthephenylpropanoid D/E were first discovered and obtained in potato [39]. Interestingly, *Solanum tuberosum* L. and *Lycium ruthenicum* Murr. Belong to the Solanaceae Juss. in which the major anthocyanins are all petanin, and norpetanin also was purified from *Lycium ruthenicum* (Unpublished work from our research group). Consequently, these results may imply novel degradation or metabolic pathways of anthocyanins, such as from petanin to norpetanin to lyciruthephenylpropanoid D/E to 4-*O*-(*p*-coumaryl) rhamnose, and need to be supported by more direct evidence.

### 2.2. Screening and Antimelanogenesis of Tyrosinase Inhibitors

**Figure 3:**
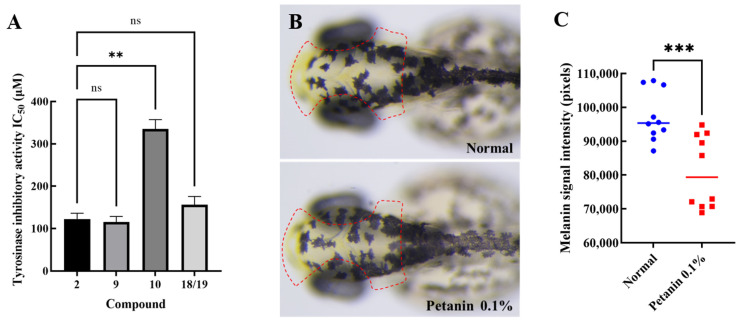
Tyrosine inhibitory activity and anti-melanogenesis of anthocyanins and their novel degradation compounds (**A**) Tyrosine inhibits activity (**: *p* < 0.01, ns: not significant); (**B**) Melanin in zebrafish (Red box region is the characteristic site of melanin production in zebrafish); (**C**) Melanin signal intensity in zebrafish (***: *p* < 0.001).

Anthocyanins and their novel degradation compounds (**1**–**19**) were tested for tyrosinase inhibitory activity using a tyrosinase inhibitor screening kit. As shown in Table 4 and Figure 3A, petanin, norpetanin, 4-*O*-(*p*-coumaryl) rhamnose, and lyciruthephenylpropanoid D/E possessed tyrosinase inhibitory activity with IC_50_ values of 122.37 ± 8.03, 115.53 ± 7.51, 335.03 ± 12.99, and 156.27 ± 11.22 μM (Mean ± SEM, *n* = 3), respectively. However, the IC_50_ of kojic acid, which was lower than that of petanin and its degradation compounds, was 20.58 ± 1.62 μM. In addition, the tyrosinase inhibitory effect of other compounds (0.60 mM) were shown in Table S2.

Petanin, a major compound in purple potato, was tested for its safety and anti-melanogenic effects in zebrafish, as shown in Table S3 and Figure 3B,C.

Anthocyanins purified from *Lycium ruthenicum* Murr. had inhibitory effect on tyrosinase monophenolase (IC_50_ = 1.483 ± 0.058 mg mL^−1^), and the maximum inhibitory activity of the purified anthocyanins (3.00 mg mL^−1^) on tyrosinase diphenolase was 42.16% ± 0.77% [28]. Petunidin 3-O-glucoside may act as a tyrosinase inhibitor to block melanin production, and its IC_50_ value was 10.3 ± 1.0 µM [29]. As shown in Table 4 and Figure 3A, regarding the tyrosinase activity of petanin and its degradation compounds, petanin and 4-*O*-(*p*-coumaryl) rhamnose were shown to be significantly different (*p* < 0.01). However, there was no significant difference between petanin, norpetanin, and lyciruthephenylpropanoid D/E (*p* > 0.05). Therefore, the acyl group containing two sugar groups may be the ‘key’ to the ‘lock’ for tyrosinase, whereas 4-*O*-(*p*-coumaryl) rhamnose may not have the same effect because the molecule is too small. In fact, according to the results of the evaluation of toxicity and anti-melanogenic effects in zebrafish, petanin not only had a very safe tolerated dose (MTC = 0.15%), but also showed a significant anti-melanogenic effect (17%, *p* < 0.05) at a concentration of 0.1% compared with the normal group, as shown in Figure 3B,C. Ultimately, petanin and its analogues inhibited tyrosinase activity, and its parent nucleus and acyl group may be important active groups.

### 2.3. Molecular Docking and Dynamic Simulation of Tyrosinase Inhibitors

**Figure 4:**
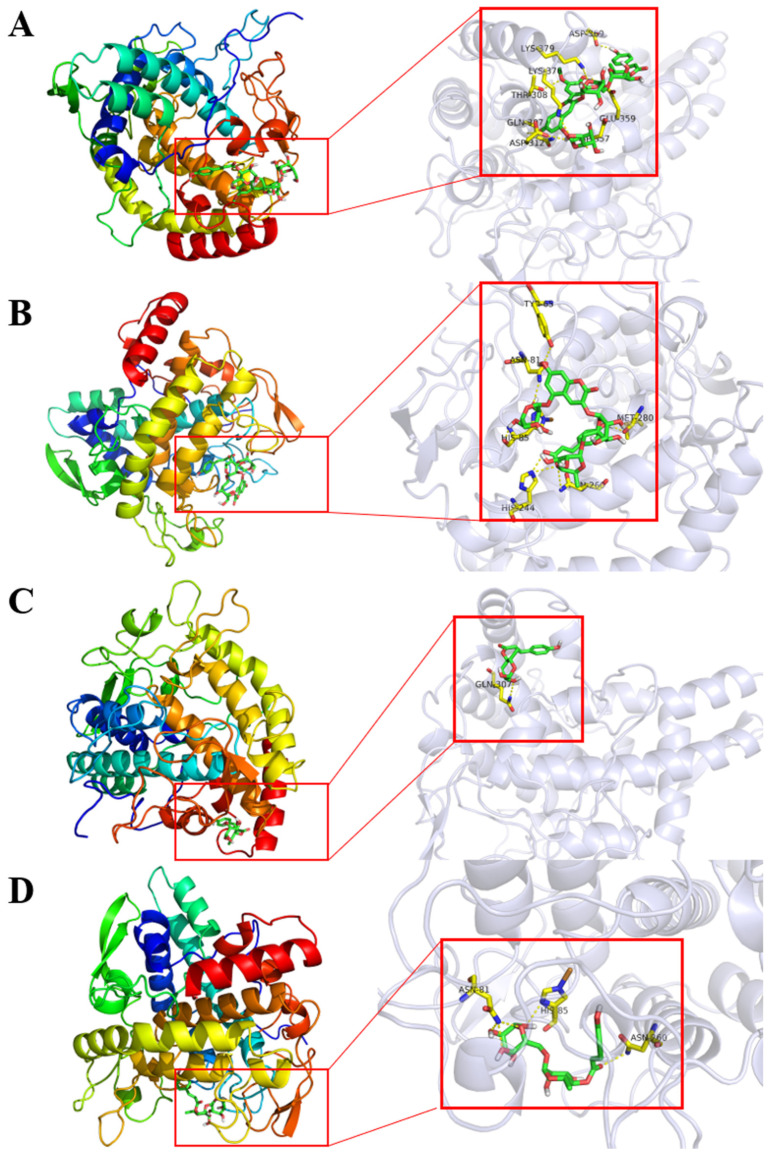
The 3D intermolecular interactions for the docked complexes of tyrosinase with: (**A**) petanin, (**B**) norpetanin, (**C**) 4-*O*-(*p*-coumaryl) rhamnose, and (**D**) Lyciruthephenylpropanoid D/E.

Evaluation of petanin and its degradation compounds were performed against tyrosinase using AutoDock Vina. They depicted considerable docking energy and formation of intermolecular interactions with the essential residues of tyrosinase enzyme in the respective docked complexes (Figure 4 and Table 4). In addition, the ADMET of petanin was similar to that of norpetanin, as shown in Figure S18.

**Figure 5:**
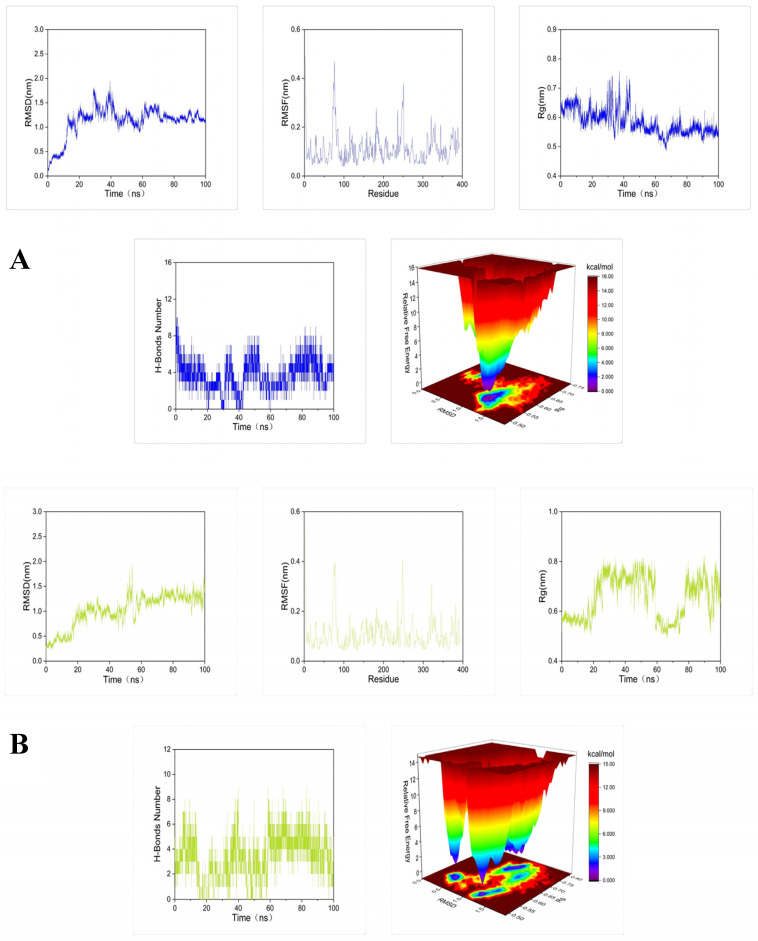

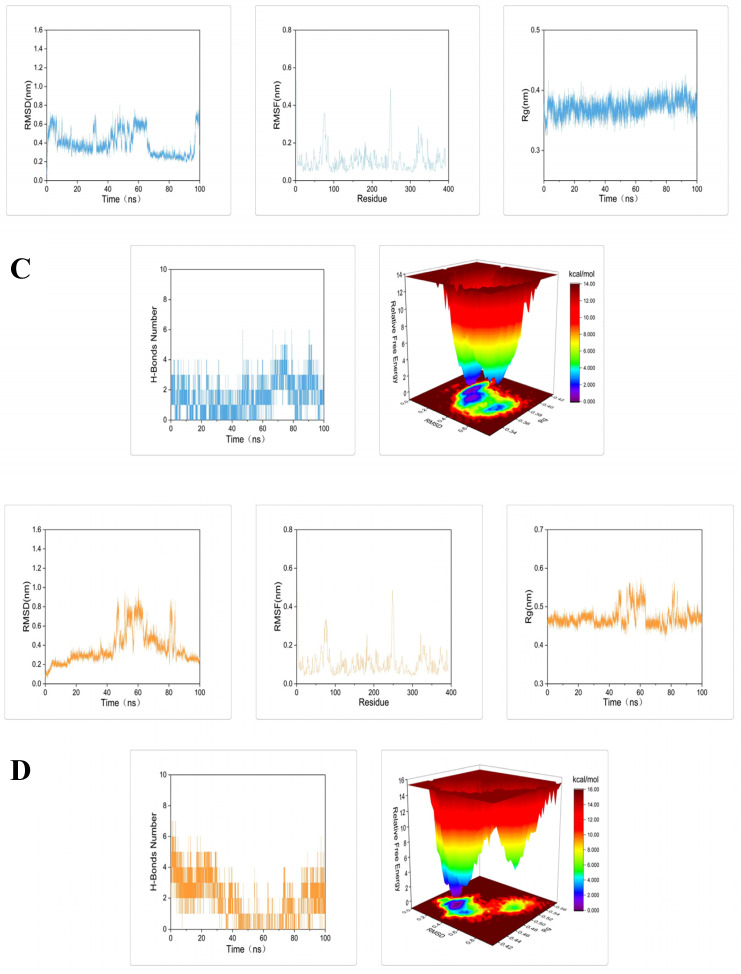
Molecular dynamics simulations of 100 ns for tyrosinase docked with: (**A**) petanin, (**B**) norpetanin, (**C**) 4-*O*-(*p*-coumaryl) rhamnose, and (**D**) Lyciruthephenylpropanoid D/E.

To further demonstrate the degree and stability of the binding between the compound and the protein, molecular dynamics simulations of 100 ns were performed for each set of docking results. The root mean square deviation (RMSD), root mean square fluctuation (RMSF), radius of gyration (Rg), number of hydrogen bonds, and Gibbs free energy plots were analyzed in each set of molecular dynamics simulation trajectories (Figure 5).

Molecular docking results showed that petanin and its degradation compounds had a huge binding energy with tyrosinase, but 4-*O*-(*p*-coumaryl) rhamnose had smaller binding energies and fewer hydrogen bonds than the other compounds, as shown in Table 4 and Figure 4. However, hydrogen bonding and hydrophobic interactions are well established as essential factors in the stability of ligands at the active pocket of receptors [41]. Thus, the differences observed in the tyrosinase inhibitory activity of petanin and its degradation compounds may be related to the number and position of hydrogen bonds they form with tyrosinase.

Results of root mean square deviation (RMSD), root mean square fluctuation (RMSF), radius of gyration (Rg), number of hydrogen bonds, and Gibbs free energy plots in the molecular dynamics simulation trajectories of the complex are shown in Figure 5. The RMSD fluctuation curve of molecular dynamics simulation reached a stable state without large fluctuations within 100 ns, and the fluctuation range was within 0–0.3 nm. This indicates that these compounds can form stable complexes with tyrosinase [42]. The RMSF showed that the amino acid residues in the complex all fluctuated around the amino acid residues at positions 80 and 250. This may be a normal fluctuation caused by the binding of the component small molecules to the protein [43]. The Rg curves all fluctuated in the range of 0.1–0.2 nm, indicating that each complex formed a tight and stable complex structure [44]. Calculation of the number of hydrogen bonds between the compound and the protein showed that petanin and norpetanin had four hydrogen bonds on average, lyciruthephenylpropanoid D/E had three hydrogen bonds, and 4-*O*-(*p*-coumaryl) rhamnose had only two. The difference in the number of hydrogen bonds formed may be the main factor affecting the stability of the complex. Gibbs free energy plots showed that petanin formed the most stable complex with tyrosinase compared to its other compounds [45]. In conclusion, petanin and its degradation compounds formed stable complexes with tyrosinase, and this stability benefited from conformational fluctuations of the protein, fluctuations in the level of protein residues, protein folding, and the number of hydrogen bonds.

## 3. Materials and Methods

### 3.1. Plant Materials

*Solanum tuberosum* L. (Heijingang) fresh tubers were purchased from Yaodian Town, Dingxi City, Gansu Province, P. R. China (Latitude: 35°18′35″ N, Longitude: 104°03′23″ E, Altitude: approximately 2175 m) in June 2022, and were identified by Professor Baolong Liu of the Northwest Institute of Plateau Biology, Chinese Academy of Sciences (NWIPB, CAS). A voucher specimen (Nwipb0335504) was deposited in the herbarium of the Key Laboratory of Tibetan Medicine Research (NWIPB, CAS).

### 3.2. General Experimental Procedures

The ^1^H (600 MHz/800 MHz) and ^13^C (600 MHz/800 MHz) NMR spectra were recorded using a Bruker AVANCE III 600 MHz Spectrometer (^1^H NMR spectra parameter configuration: NS = 4, DS = 2, DW = 41.6 usec. ^13^C NMR spectra parameter configuration: NS = 4/1024, DS = 2/4, DW = 41.6/13.8 usec.) and Bruker AV 800MHz Spectrometer (^1^H NMR spectra parameter configuration: NS = 4, DS = 0, DW = 31.2 usec. 2D NMR spectra parameter configuration: NS = 8/16, DS = 16/16, DW = 56.8/59.4 usec) (Bruker, Billerica, MA, USA) in a deuterated solvent. HR-ESI-MS and ESI-MS analyses were performed using a Shimadzu Corporation UPLC-IT-TOF spectrometer (Shimadzu, Kyoto, Japan). Preparative high-performance liquid chromatography was performed on a DAC-HB50 separation module combined with a UV detector at 535 and 280 nm (Hanbon, Huai’an, China). A C_18_-ODS-A (300 mm × 50 mm, 5 μm, YMC, Kyoto, Japan) and XAqua C_18_ semi-preparative column (4.6 × 250 mm, 5 μm, Acchrom, Wenling, China) were used for separation. UV spectra were acquired using a T6 New Century spectrophotometer (Persee, Beijing, China). Microscopic observation and photographs were taken using a SZX7 dissecting microscope (Olympus, Tokyo, Japan) and a CCD camera (VertA1, Shanghai, China). All chemicals and solvents were of chromatographic grade.

### 3.3. Extraction and Isolation

**Figure 6:**
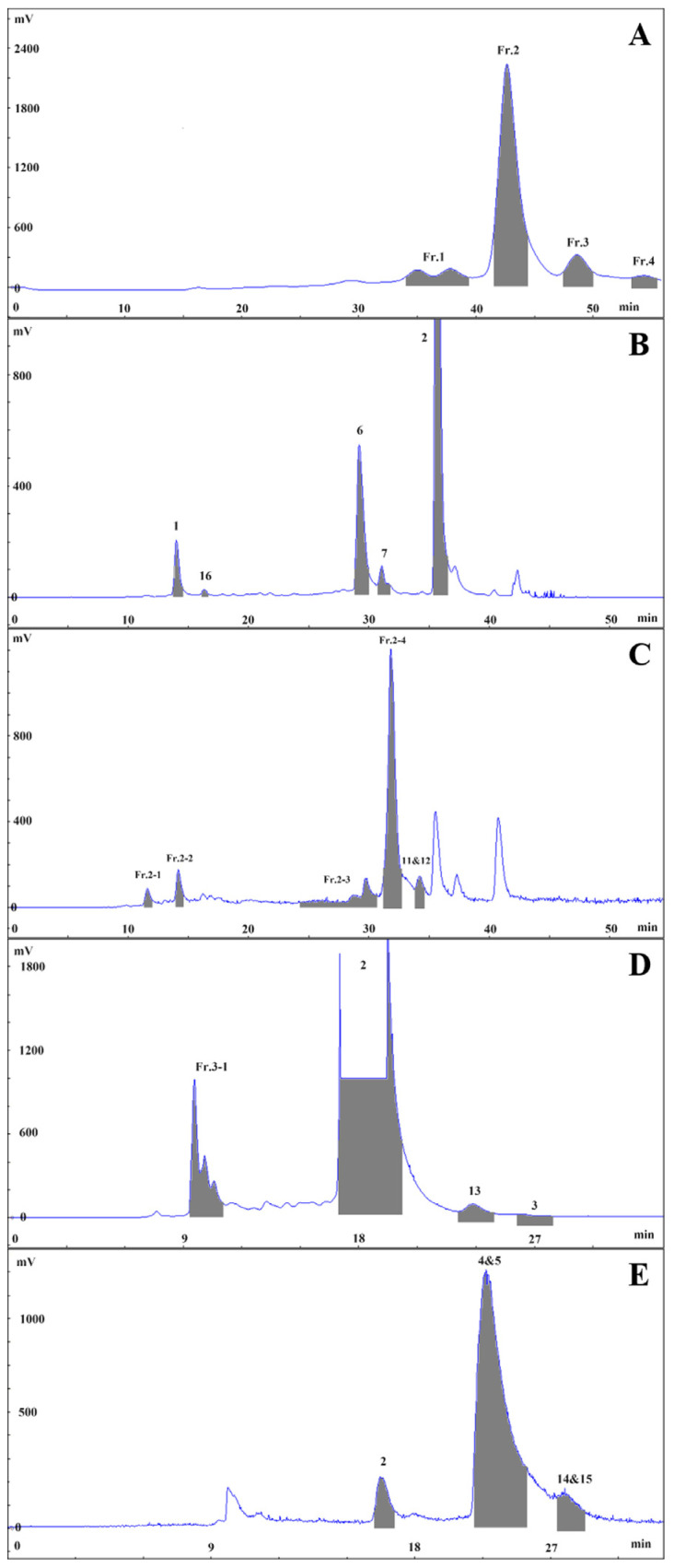
Isolation and purification chromatography of *Solanum tuberosum* L. extract (**A**), Fr.1 (**B**), Fr.2 (**C**), Fr.3 (**D**) and Fr.4 (**E**).

Fresh slices of potato tubers (100.00 kg) were subjected to extraction with 70% EtOH-Britton-Robinson buffer, and the solvent was evaporated under vacuum to obtain a crude extract (1.58 kg). The crude extract was partitioned with D101 macroporous adsorbent resin using an elution gradient of EtOH: water (0:100, 5 BV; 95:5, 4 BV) to obtain the potato extractum (100.50 g, a percentage yield of 0.10%, FW). The potato extract (40.00 g) was fractionated with DAC-HB50 using a water (0.60% trifluoroacetic acid)-acetonitrile gradient (0~55 min, 15%~35% acetonitrile; 50 mL, 535 nm) to obtain four fractions (Fr.1: 33.2~38.6 min, Fr.2: 40.6~43.5 min, Fr.3: 46.4~48.9 min, Fr.4: 52.8~54.6 min), as shown in Figure 6A. These fractions were separated using an XAqua C_18_ semi-preparative column with a water (0.6% trifluoroacetic acid)-acetonitrile elution system and various elution gradients in Fr.3, Fr.4, Fr.1, and Fr.2, as shown in Figure 6B–E.

#### 3.3.1. Isolation and Purification of Fr.3

Fr.3 was eluted (0~35 min, 23%~23% acetonitrile; 8 mL, 535 nm) to give Fr.3-1 (9.2~11.1 min), **2** (16.9~20.2 min, 17.4 mg), **13** (23.1~25.0 min, 4.7 mg) and **3** (26.1~27.9 min, 5.2 mg). Then, Fr.3-1 was purified (0~40~45~55 min, 5%~20%~95%~95% acetonitrile; 8 mL, 535 nm) to yield **1** (33.2~34.2 min, 5.3 mg) and **16** (42.5~43.3 min, 2.6 mg).

#### 3.3.2. Isolation and Purification of Fr.4

Fr.4 was eluted (0~30 min, 25~25% acetonitrile; 8 mL, 535 nm) to give **2** (16.1~16.9 min), **4**, and **5** (20.5~22.7 min, 46.6 mg), along with **14** and **15** (24.1~25.3 min, 2.8 mg).

#### 3.3.3. Isolation and Purification of Fr.1

Fr.1 was eluted (0~40~45~55 min, 15%~25%~95%~95% acetonitrile; 8 mL, 535 nm) to give **1** (13.9~14.9 min), 16 (16.4~16.9 min), **6** (28.9~30.2 min, 11.1 mg), **7** (31.0~32.1 min, 14.5 mg), and **2** (35.4~36.8 min).

#### 3.3.4. Isolation and Purification of Fr.2

Fr.2 was eluted (0~50~55 min, 15%~27.5%~95% acetonitrile; 8 mL, 535 nm) to give Fr.2-1 (11.4~12.1 min), Fr.2-2 (13.9~14.6 min), Fr.2-3 (24.3~30.7 min), Fr.2-4 (31.4~32.8 min), and **11** and **12** (33.9~34.9 min, 4.9 mg). Then, Fr.2-1 was purified (0~20~40 min, 5%~11.5%~18% acetonitrile; 8 mL, 535 nm) to yield **8** (31.5~32.5 min, 2.4 mg). Fr.2-2 was purified (0~20~40~50 min, 5%~11.5%~18%~21.5% acetonitrile; 8 mL, 535 nm) to yield **1** (35.2~36.2 min). Fr.2-3 was purified (0~100 min, 16%~16% acetonitrile, 8 mL, 280 nm) to yield **18** and **19** (40.4~45.6 min, 44.3 mg), **10** (50.8~56.3 min, 2.1 mg), **9** (81.5~86.5 min, 29.4 mg), and **17** (89.9~93.7 min, 1.6 mg). Finally, Fr.2-4 was purified (0~40 min, 18%~18% acetonitrile, 8 mL, 535 nm) to yield **7** (23.7~27.2 min).

### 3.4. Screening and Validation of Inhibitory Tyrosinase Activity

#### 3.4.1. Screening for Tyrosinase Inhibitory Activity

Tyrosinase inhibitory activity was determined using a MAK257-1K tyrosinase inhibitor screening kit (Colorimetric; Sigma-Aldrich, Shanghai, China) (Lot: 7J08K05750), according to the manufacturer’s instructions. All samples were dissolved into the water (a 1.0 mM stock solution) and diluted to the proper test concentration with tyrosinase assay buffer before use. All tests were performed thrice independently, including inhibitor control (kojic acid) at an inhibitory ratio at 0.60 mM. The IC_50_ of petanin (**2**) and its derivatives (**9** Norpetanin, **10** 4-*O*-(*p*-coumaryl) rhamnose, and **18** and **19** Lyciruthephenylpropanoid D/E) was calculated using GraphPad Prism (9.5.0, GraphPad Software, Boston, MA, USA).

#### 3.4.2. Safety Evaluation and Verification of Anti-Melanogenic Effect

Wild-type AB strain zebrafish were used for safety evaluation in the experimental system. The age of zebrafish was 6 h after fertilization (6 hpf). The sample size of each group was 30 tails (*n* = 30). The adult fish were raised and bred according to the laboratory standards and in accordance with the requirements of the International AAALAC certification (Certification number: 001458). The experimental protocol was as follows: 1. The zebrafish were randomly selected in six-well plates, with 30 fish in each well. 2. Samples were dissolved in water, and the normal group and petanin 0.1% group were set up at the same time. The volume of each well was 3 mL. 3. The cells were incubated at 28 °C for 45 h in the dark. 4. The maximum tolerated concentration (MTC) of samples was determined for normal zebrafish based on zebrafish death count, death rate, and toxicity.

The anti-melanogenesis effect of petanin was also tested in zebrafish. The experimental protocol was as follows: 1. The zebrafish were randomly selected in six-well plates, with 30 fish in each well. 2. Samples were dissolved in water, and the normal group and petanin 0.1% group were set up at the same time. The volume of each well was 3 mL. 3. The cells were incubated at 28 °C for 45 h in the dark. 4. Ten zebrafish in each experimental group (*n* = 10) were randomly selected and photographed under a microscope, and the data were analyzed and collected by ImageJ. According to the Formula (1), the melanin inhibition rate of each sample was calculated and judged.

(1) $$\mathrm{Melanin}\;\mathrm{inhibition}\;\mathrm{rate}\;(\%)=\frac{S\;(Normal)-S\;(Petanin\;0.1\%)}{S(Normal)}*100\%,$$

### 3.5. Molecular Docking and Dynamics Simulation

#### 3.5.1. Molecular Docking

Petanin and its derivatives (**9** Norpetanin, **10** 4-*O*-(*p*-coumaryl) rhamnose, and **18** and **19** Lyciruthephenylpropanoid D/E) were docked with tyrosinase (2y9x, from Protein Data Bank, www.rcsb.org) to analyze their binding affinities. The software packages used were as follows: ChemDraw (20.0.0.41) and Chem3D (20.0.0.41) for compounds structure, PyMOL (3.0.0) for ligands, and AutoDock Tools (1.5.6) and AutoDock Vina (1.2.5) for molecular docking. The ADMET of petanin and its derivatives were predicted by using the SwissADME (http://www.swissadme.ch/).

#### 3.5.2. Dynamics Simulation

Gromacs (2020.3) software was used to simulate the molecular dynamics of the protein ligand complexes obtained via molecular docking. Charmm36 was selected as the protein force field, and Gaff2 was selected as the ligand force field. A TIP3P water model was used to add solvent to the protein ligand system, and a water box with a periodic boundary of 1.2 nm was established. Prior to formal dynamics simulations, the complex was subjected to energy minimization for 50,000 steps using conjugate gradient algorithms, followed by a further balancing system of 100 ps using an isothermal (310 K) system (NVT) and an isobaric (one standard atmosphere) system (NPT), and finally a molecular dynamics simulation at room temperature and pressure for 100 ns. The root mean square deviation (RMSD), root mean square fluctuation (RMSF), radius of gyration (Rg), number of hydrogen bonds, and Gibbs free energy plots for each set of molecular dynamics simulation trajectories were analyzed.

### 3.6. Statistical Analysis

The comparison of means between different groups of numerical variables was performed using a one-way ANOVA or *t* test. A *p* value less than 0.05 (*p* < 0.05) was considered to be statistically significant.

## 4. Conclusions

In this study, six petunidin anthocyanins were isolated and purified from fresh purple potato, together with four peonidin anthocyanins, three malvidin anthocyanin, and two delphinidin anthocyanins. These were mainly acylated anthocyanins. The novel compounds isolated and identified, norpetanin and 4-*O*-(*p*-coumaryl) rhamnose, may indicate new metabolic pathways for anthocyanins: rearrangement or degradation at the B-ring, such as is seen in the pathway from petanin to norpetanin. However, more evidence is needed to support and elucidate the mechanism of the degradation or rearrangement reactions of anthocyanins. Moreover, petanin and its degradation compounds (such as norpetanin and lyciruthephenylpropanoid D/E) showed promising potential as tyrosinase inhibitors based on both experimental and molecular docking and dynamic simulations. Hence, the chemical composition and biological activities of purple potato are now more widely recognized and understood, which facilitates the wider development and utilization of this crop, such as in the treatment of diseases related to melanin production and the development of cosmetic products with whitening efficacy. However, the evidence supporting the degradation pathway of petanin in purple potato is thus far very scarce and has not been clearly demonstrated, and transcriptomics and metabolomics should be investigated to provide more favorable evidence in future.

## Figures and Tables

**Table 1:** The anthocyanins composition of purple potato skins.

No.	Compound Name	Content Range (mg g^−1^)
1	petunidin 3-*O*-*p*-coumaroylrutinoside-5-*O*-glucoside	0.282~0.843
2	petunidin 3-*O*-feruloylrutinoside-5-*O*-glucoside	0.0963~0.269
3	pelargonidin 3-*O*-*p*-coumaroylrutinoside-5-*O*-glucoside	0~0.0387
4	peonidin 3-*O*-*p*-coumaroylrutinoside-5-*O*-glucoside	0~0.241
5	malvidin 3-*O*-*p*-coumaroylrutinoside-5-*O*-glucoside	0~0.656
6	peonidin 3-*O*-feruloylrutinoside-5-*O*-glucoside	0~0.218
7	malvidin 3-*O*-feruloylrutinoside-5-*O*-glucoside	0~0.212
8	petunidin 3-*O*-rutinoside-5-*O*-glucoside	0.510~1.34
9	petunidin 3-*O*-rutinoside-5-*O*-rhamnoside	0~1.13
10	petunidin 3-*O*-caffeoylrutinoside-5-*O*-glucoside	0~1.07
11	petunidin 3-*O*-*p*-coumaroylrutinoside-5-*O*-glucoside	1.12~2.08
12	petunidin 3-*O*-feruloylrutinoside-5-*O*-glucoside	0.520~1.13
14	petunidin 3-*O*-*p*-coumaroylrutinoside	0~0.400

**Table 2:** The ^1^H and ^13^C NMR spectral data for **9** in CD_3_OD/CF_3_COOD (9:1).

No.	^1^H NMR	^13^C NMR	No.	^1^H NMR	^13^C NMR
2	―	160.4 (s)	3′′	3.90 (1H, overlapped)	70.3 (d)
3	―	139.0 (s)	4′′	4.94 (1H, t-like, 9.5)	75.3 (d)
4	7.69 (1H, s)	117.2 (d)	5′′	3.82 (1H, dq, 9.5, 6.2)	67.6 (d)
5	―	155.4 (s)	6′′	1.00 (3H, d, 6.2)	17.8 (q)
6	6.60 (1H, d, 1.4)	101.2 (d)	Glc-1′′′	4.95 (1H, d, 7.8)	102.7 (d)
7	―	161.4 (s)	2′′′	3.57 (1H, dd, 8.5, 7.8)	74.9 (d)
8	6.37 (1H, d, 1.4)	97.6 (d)	3′′′	3.48 (1H, overlapped)	78.3 (d)
9	―	153.3 (s)	4′′′	3.49 (1H, overlapped)	71.0 (d)
10	―	104.4 (s)	5′′′	3.51 (1H, m)	77.5 (d)
Glc-1′	5.07 (1H, d, 7.0)	101.8 (d)	6′′′	3.74 (1H, br d, 12.1) 3.88 (1H, overlapped)	62.2 (t)
2′	3.51 (1H, overlapped)	74.4 (d)	1′′′′	―	127.2 (s)
3′	3.49 (1H, overlapped)	78.1 (d)	2′′′′, 6′′′′	7.48 (2H, d, 8.4)	131.3 (d)
4′	3.41 (1H, t-like, 8.6)	71.3 (d)	3′′′′, 5′′′′	6.80 (2H, d, 8.4)	116.8 (d)
5′	3.69 (1H, m)	76.9 (d)	4′′′′	―	161.3 (s)
6′	3.69 (1H, overlapped) 4.01 (1H, br d, 9.6)	67.0 (t)	7′′′′	7.60 (1H, d, 15.9)	147.0 (d)
Rha-1′′	4.76 (1H, br s)	101.7 (d)	8′′′′	6.34 (1H, d, 15.9)	115.1 (d)
2′′	3.88 (1H, br s)	72.1 (d)	9′′′′	―	169.1 (s)

**Table 3:** The ^1^H and ^13^C NMR spectral data for **10** in CD_3_OD.

No.	^1^H NMR	^13^C NMR
Rha-1	5.04 (1H, br s)	95.8 (d)
2	3.83 (1H, br s)	73.1 (d)
3	3.93 (1H, dd, 9.7, 2.9)	70.3 (d)
4	5.02 (1H, t-like, 9.7)	75.7 (d)
5	4.01 (1H, dq, 9.7, 6.3)	67.3 (d)
6	1.14 (3H, d, 6.3)	18.0 (q)
1′	―	127.2 (s)
2′, 6′	7.47 (2H, d, 8.5)	131.2 (d)
3′, 5′	6.80 (2H, d, 8.5)	116.8 (d)
4′	―	161.3 (s)
7′	7.64 (1H, d, 15.9)	146.8 (d)
8′	6.36 (1H, d, 15.9)	115.2 (d)
9′	―	169.0 (s)

**Table 4:** Tyrosinase inhibitory activity (IC_50_), affinity, and amino acids of petanin and its degradation compounds.

NO.	Name	IC_50_ (μM)	Affinity (kcal/mol)	Amino Acids
**2**	Petanin	122.37 ± 8.03	−8.5	GLN-307/THR-308/ASP-312/ASP-357/GLU-359/LYS-369/LYS-376/LYS-379
**9**	Norpetanin	115.53 ± 7.51	−8.5	TYR-65/ASN-81/HIS-85/HIS-244/ASN-260/MET-280
**10**	4-O-(p-coumaryl) rhamnose	335.03 ± 12.99	−7.6	GLN-307
**18** and **19**	Lyciruthephenylpropanoid D/E	156.27 ± 11.22	−8.4/−8.5	ASN-81/HIS-85/ASN-260

## Data Availability

Data are contained within the article and supplementary materials.

## Supplementary Materials

The following supporting information can be downloaded at: https://www.mdpi.com/article/10.3390/molecules29071492/s1, Figure S1: ^1^H NMR spectrum (600 MHz, CD_3_OD/CF_3_COOD (9:1)) of **9**; Figure S2: ^13^C NMR spectrum (600 MHz, CD_3_OD/CF_3_COOD (9:1)) of **9**; Figure S3: HMBC spectrum and key HMBC correlations of **9**; Figure S4: HSQC spectrum of **9**; Figure S5: ^1^H, ^1^H-COSY spectrum and key ^1^H, ^1^H-COSY correlations of **9**; Figure S6: HR-ESI-MS spectrum of **9**; Figure S7: ^1^H NMR spectrum (800 MHz, CD_3_OD) of **10**; Figure S8: ^13^C NMR spectrum (800 MHz, CD_3_OD) of **10**; Figure S9: HMBC spectrum and key HMBC correlations of **10**; Figure S10: HSQC spectrum of **10**; Figure S11: ^1^H, ^1^H-COSY spectrum and key ^1^H, ^1^H-COSY correlations of **10**; Figure S12: ^1^H NMR spectrum (600 MHz, CD_3_OD/CF_3_COOD (9:1)) of **3**; Figure S13: ^1^H NMR spectrum (600 MHz, CD_3_OD/CF_3_COOD (9:1)) of **12**; Figure S14: ^1^H NMR spectrum (600 MHz, CD_3_OD/CF_3_COOD (9:1)) of **15**; Figure S15: HPLC chromatography of *Solanum tuberosum* L. extract (A) and Fr.1~Fr.4 (B~E); Figure S16: HPLC chromatography and spectrogram of **1**~**9**; Figure S17: HPLC chromatography and spectrogram of **10**~**19**; Figure S18: ADMET of (A) petanin, (B) norpetanin, (C) 4-*O*-(*p*-coumaryl) rhamnose, (D) Lyciruthephenylpropanoid D/E; Table S1: Retention time, purity and λ_max_ of anthocyanins and their degradation products by HPLC; Table S2: Tyrosinase inhibitory activity of anthocyanins and their degradation products (0.60 mM); Table S3: Maximum tolerated concentration of petanin in zebrafish (*n* = 30).
