# Supplementary material for: Isolation, Purification and Tyrosinase Inhibitory Activity of Anthocyanins and Their Novel Degradation Compounds from Solanum tuberosum L
Source: Molecules. 2024 Mar 27;29(7):1492. doi: 10.3390/molecules29071492 (PMC11013068; doi:10.3390/molecules29071492)
Supplement: Supplementary file 1 [file molecules-29-01492-s001.zip › molecules-2923469-supplementary.pdf]

SUPPLEMENTARY MATERIAL

Isolation, purification and tyrosinase inhibitory activity of anthocyanins and their novel degradation compounds from *Solanum tuberosum* L.

Jian Ouyang [1,2,3], Na Hu [1] and Honglun Wang [1,2,*]

[1] Qinghai Provincial Key Laboratory of Tibetan Medicine Research and CAS Key Laboratory of Tibetan Medicine Research, Northwest Institute of Plateau Biology, Xining 810008, PR China; huna@nwipb.cas.cn
[2] Huzhou China-Science Innovation Centre of Plateau Biology, Huzhou 313000, PR China; ygzjj@126.com
[3] University of Chinese Academy of Science, Beijing 100049, PR China；hlwang@nwipb.cas.cn
* Correspondence: hlwang@nwipb.cas.cn

Abstract: To explore the composition of anthocyanins and expand their biological activities, anthocyanins were systematically isolated and purified from tubers of *Solanum tuberosum* L., and their tyrosinase inhibitory activity was investigated. In this study, two new anthocyanin degradation compounds, norpetanin (**9**) and 4-*O*-(*p*-coumaryl) rhamnose (**10**), along with 17 known anthocyanins and their derivatives, were isolated and purified from an acid-ethanolic extract of fresh purple potato tubers. Their structures were elucidated via 1D and 2D NMR and HR-ESI-MS and compared with those reported in the literature. The extracts were evaluated for anthocyanins and their derivatives using a tyrosinase inhibitor screening kit and molecular docking technology, and the results showed that petanin, norpetanin, 4-*O*-(*p*-coumaryl) rhamnose, and lyciruthephenylpropanoid D/E possessed tyrosinase inhibitory activity, with 50% inhibiting concentration (IC_{50}) values of 122.37 ± 8.03, 115.53 ± 7.51, 335.03 ± 12.99, and 156.27 ± 11.22 μM (Mean ± SEM, n = 3), respectively. Furthermore, petanin was validated against melanogenesis in zebrafish; it was found that it could significantly inhibit melanin pigmentation ($p < 0.001$), and the inhibition rate of melanin was 17% compared with the normal group. This finding may provide potential treatments for diseases with abnormal melanin production, and high-quality raw materials for whitening cosmetics.

Keywords: acylated anthocyanins; norpetanin; 4-*O*-(*p*-coumaryl) rhamnose; molecular docking; petanin; tyrosinase inhibitor.

List of contents

Figure S1. ^{1}H NMR spectrum (600 MHz, CD_3OD/CF_3COOD (9:1)) of **9**

Figure S2. ^{13}C NMR spectrum (600 MHz, CD_3OD/CF_3COOD (9:1)) of **9**

Figure S3. HMBC spectrum and key HMBC correlations of **9**

Figure S4. HSQC spectrum of **9**

Figure S5. ^{1}H, ^{1}H-COSY spectrum and key ^{1}H, ^{1}H-COSY correlations of **9**

Figure S6. HR-ESI-MS spectrum of **9**

Figure S7. ^{1}H NMR spectrum (800 MHz, CD_3OD) of **10**

Figure S8. ^{13}C NMR spectrum (800 MHz, CD_3OD) of **10**

Figure S9. HMBC spectrum and key HMBC correlations of **10**

Figure S10. HSQC spectrum of **10**

Figure S11. ^{1}H, ^{1}H-COSY spectrum and key ^{1}H, ^{1}H-COSY correlations of **10**

Figure S12. ^{1}H NMR spectrum (600 MHz, CD_3OD/CF_3COOD (9:1)) of **3**

Figure S13. ^{1}H NMR spectrum (600 MHz, CD_3OD/CF_3COOD (9:1)) of **12**

Figure S14. ^{1}H NMR spectrum (600 MHz, CD_3OD/CF_3COOD (9:1)) of **15**

Figure S15. HPLC chromatography of *Solanum tuberosum* L. extract (A) and Fr.1~Fr.4 (B~E)

Figure S16. HPLC chromatography and spectrogram of **1~9**

Figure S17. HPLC chromatography and spectrogram of **10~19**

Figure S18. ADMET of (A) petanin, (B) norpetanin, (C) 4-O-(p-coumaryl) rhamnose, (D) Lyciruthephenylpropanoid D/E

Table S1. Retention time, purity and λ_{max} of anthocyanins and their degradation products by HPLC

Table S2. Tyrosinase inhibitory activity of anthocyanins and their degradation products (0.60 mM)

Table S3. Maximum tolerated concentration of petanin in zebrafish (n=30)

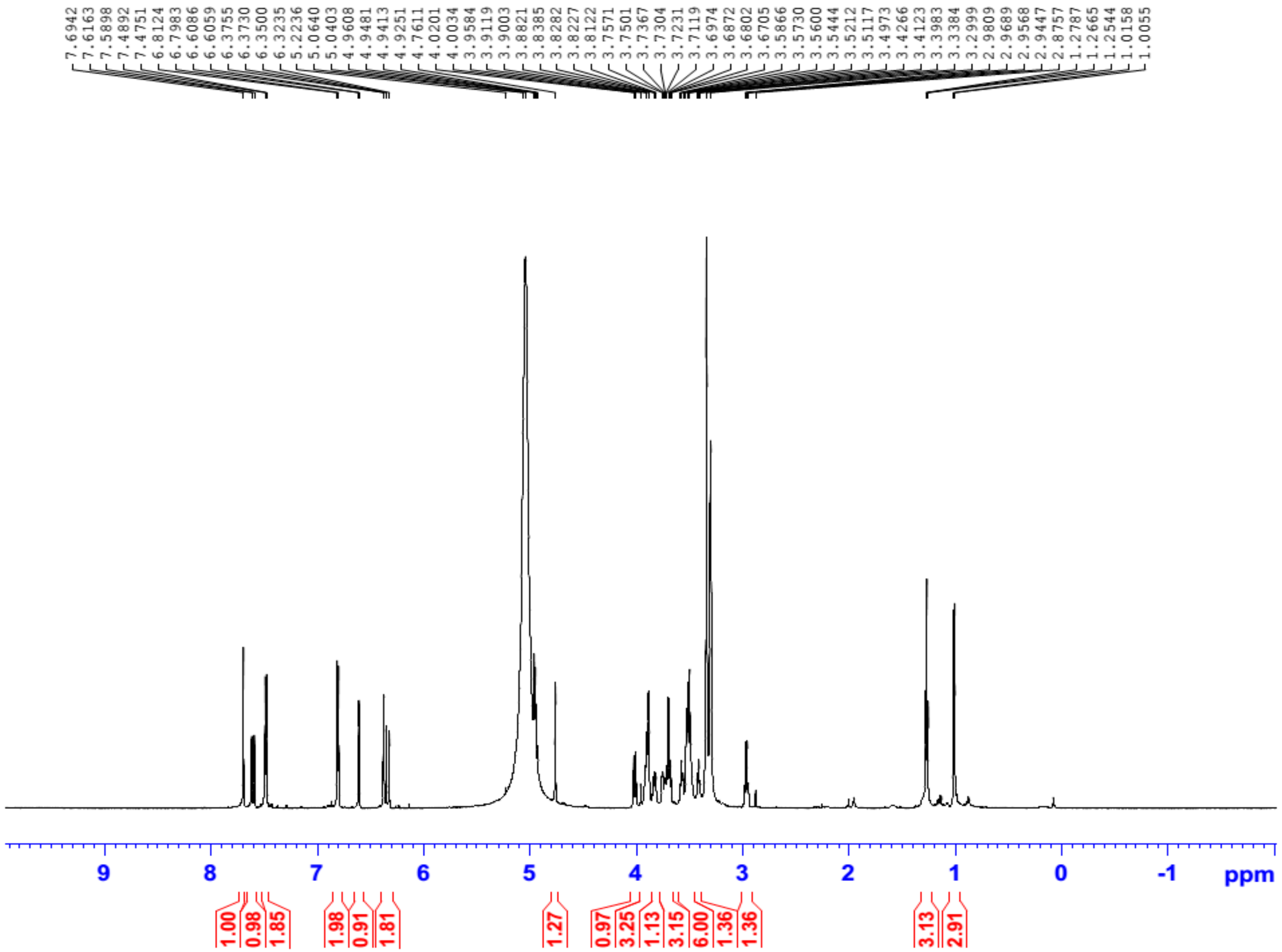

Figure S1. ^{1}H NMR spectrum (600 MHz, CD_3OD/CF_3COOD (9:1)) of **9**

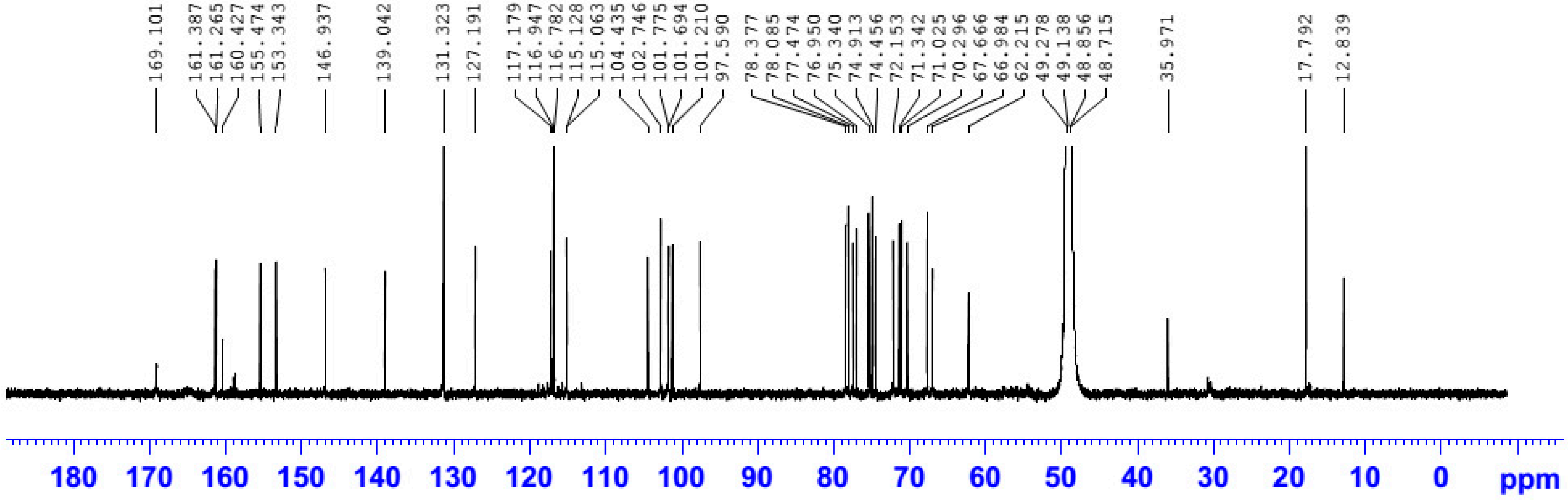

Figure S2. ^{13}C NMR spectrum (600 MHz, CD_3OD/CF_3COOD (9:1)) of **9**

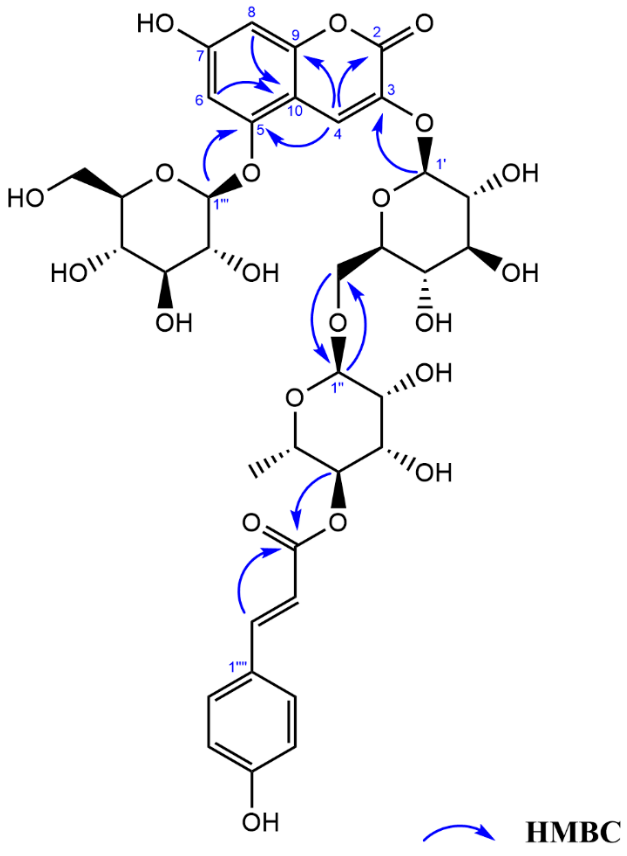

9 Norpetanin

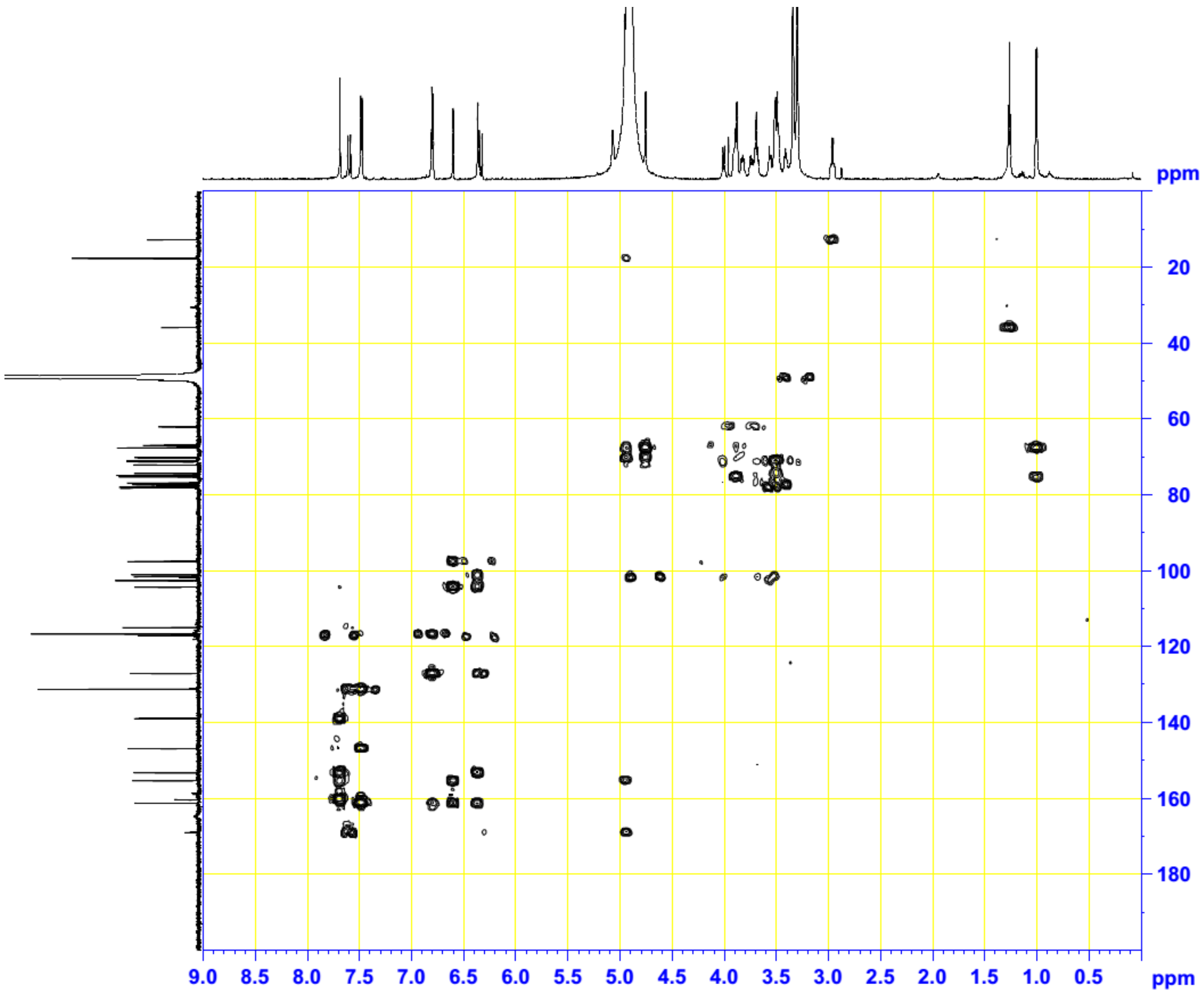

Figure S3. HMBC spectrum and key HMBC correlations of **9**

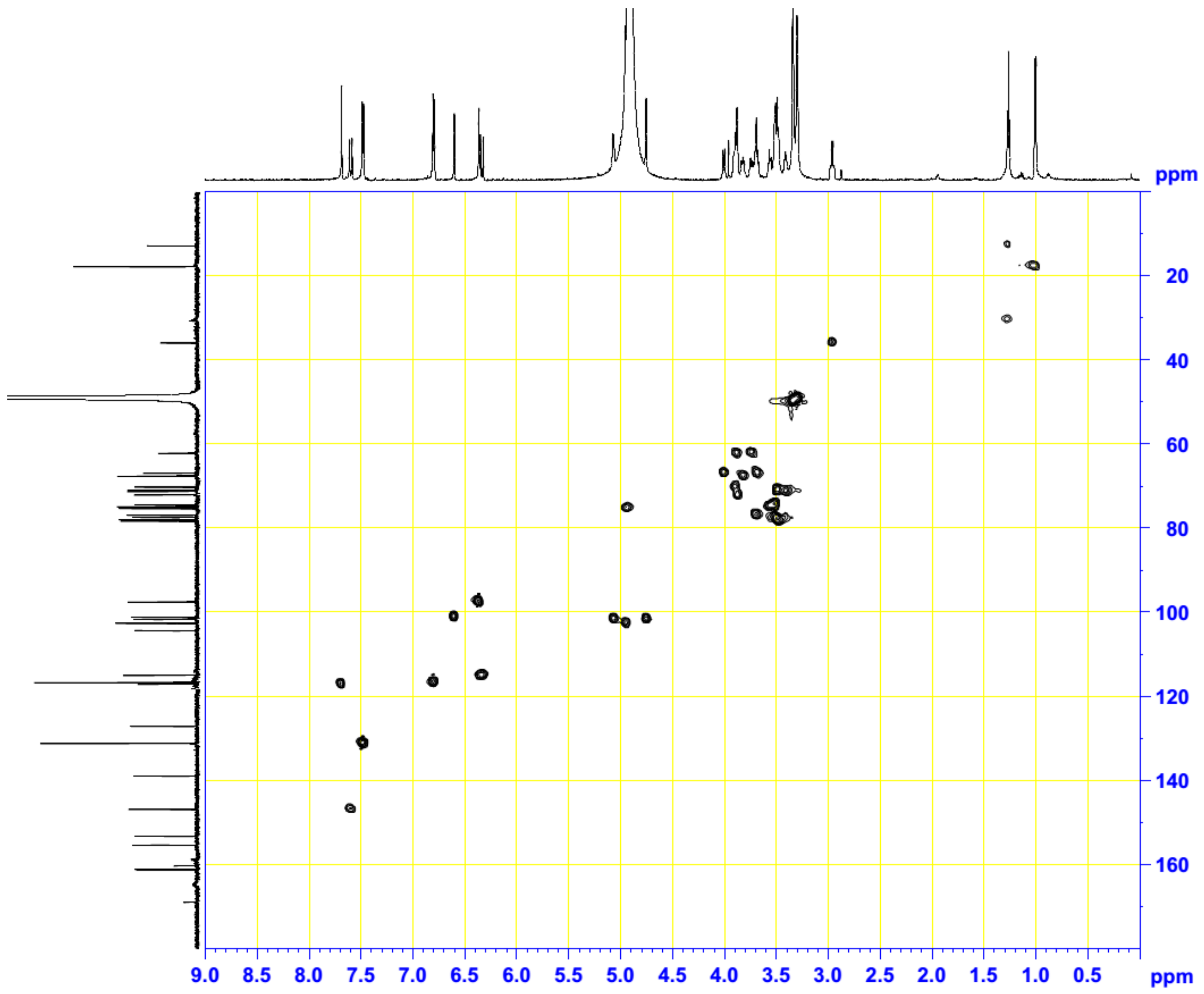

Figure S4. HSQC spectrum of **9**

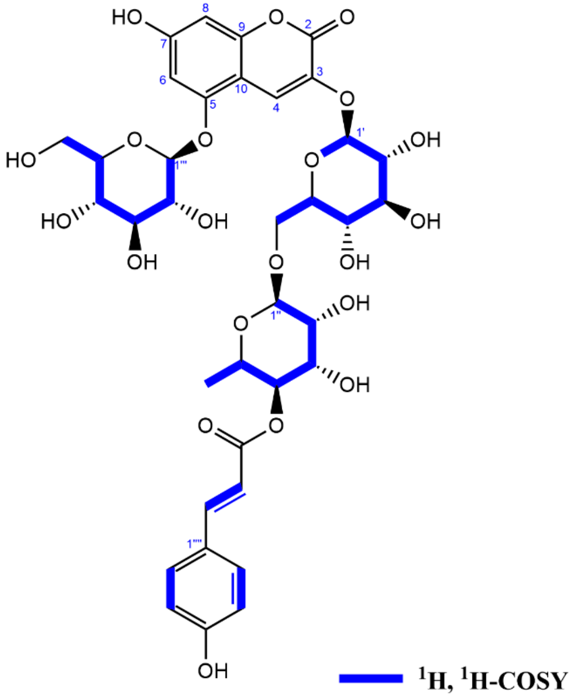

9 Norpetanin

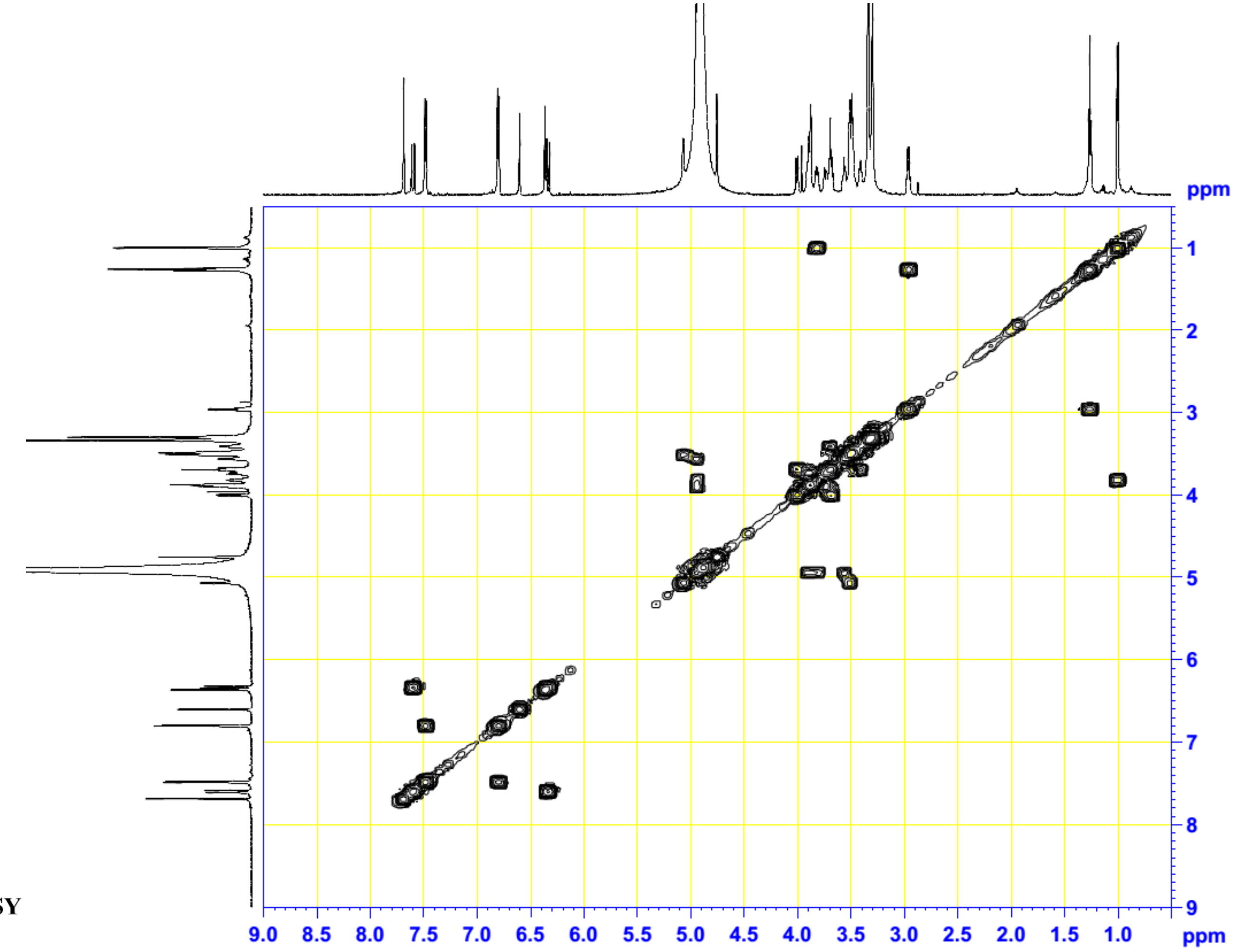

Figure S5. ^{1}H, ^{1}H-COSY spectrum and key ^{1}H, ^{1}H-COSY correlations of **9**

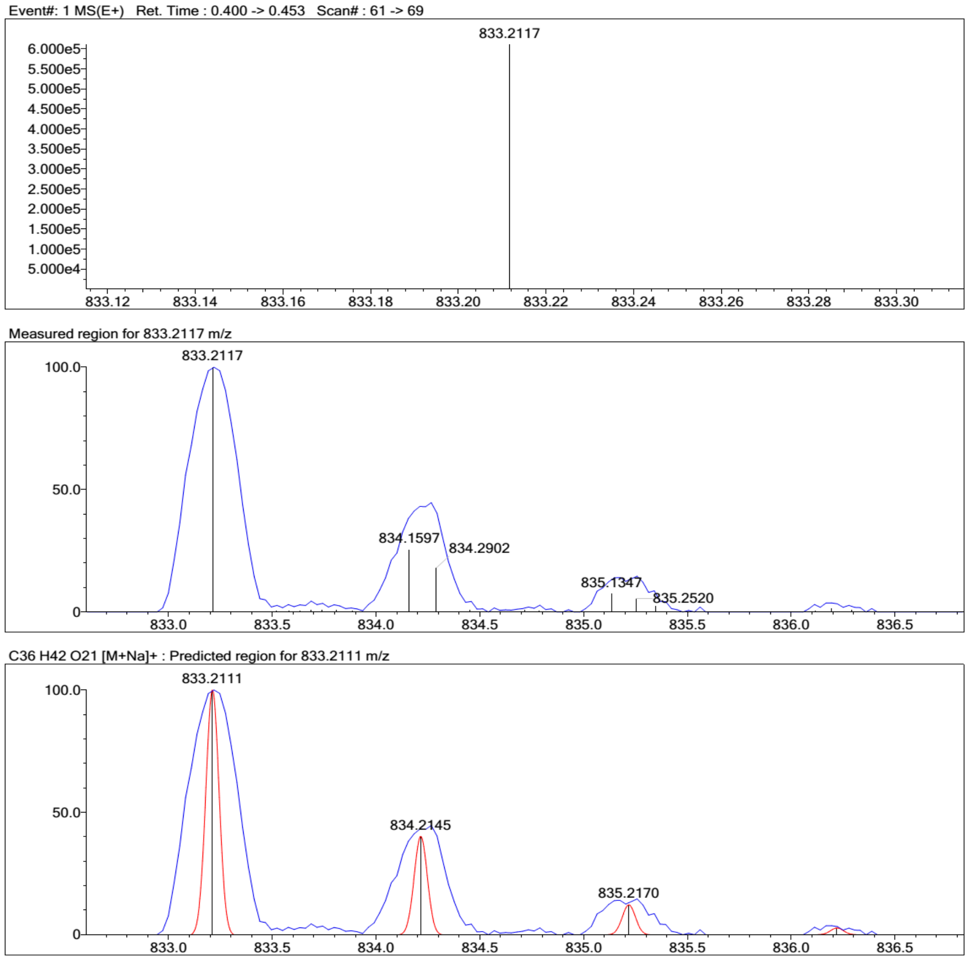

Formula (M)	Ion	Meas. m/z	Pred. m/z	Df. (mDa)	Df. (ppm)	DBE
C36 H42 O21	[M+Na]+	833.2117	833.2111	0.6	0.72	16.0

Figure S6. HR-ESI-MS spectrum of **9**

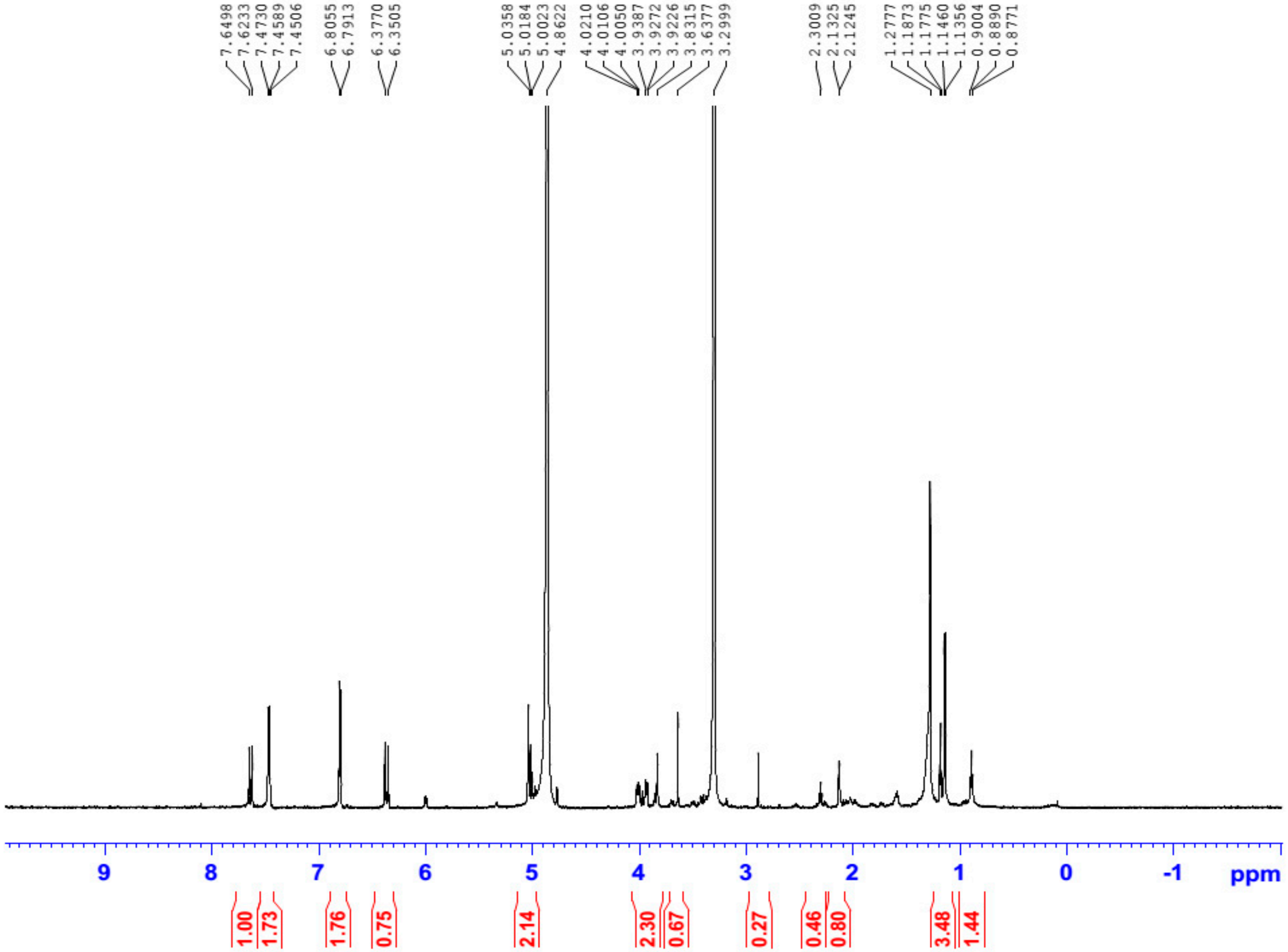

Figure S7. ^{1}H NMR spectrum (800 MHz, CD_3OD) of **10**

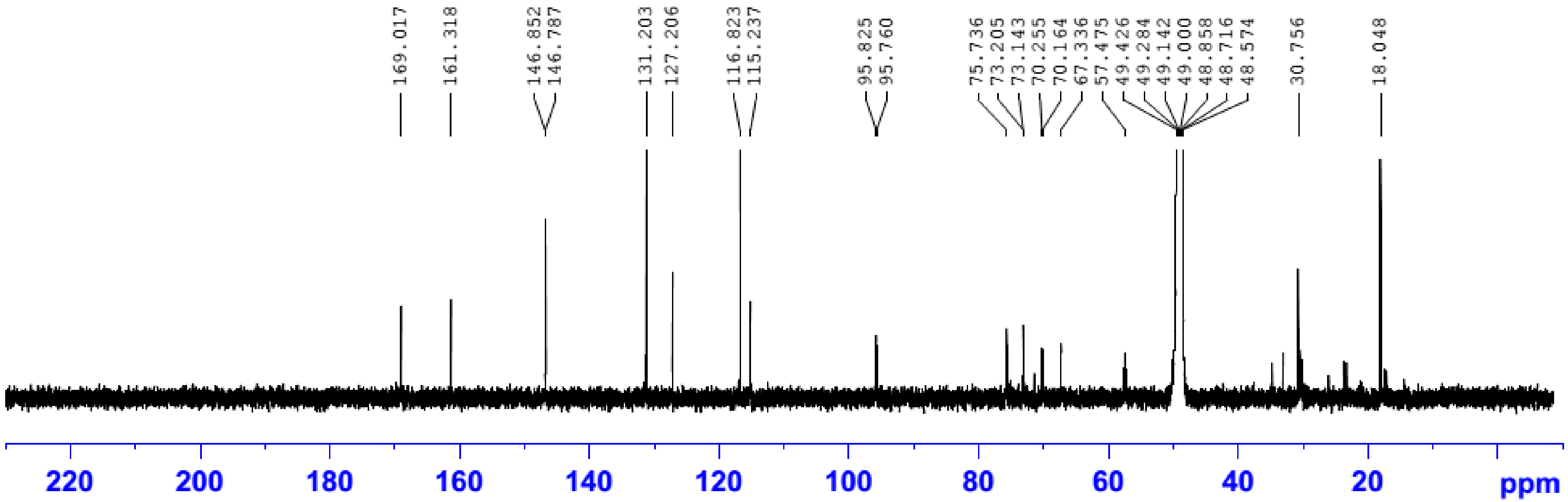

Figure S8. ^{13}C NMR spectrum (800 MHz, CD_3OD) of **10**

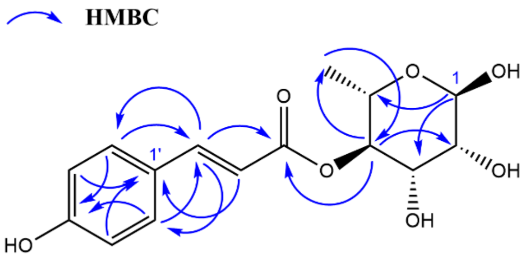

10 4-*O*-(p-coumaryl) rhamnose

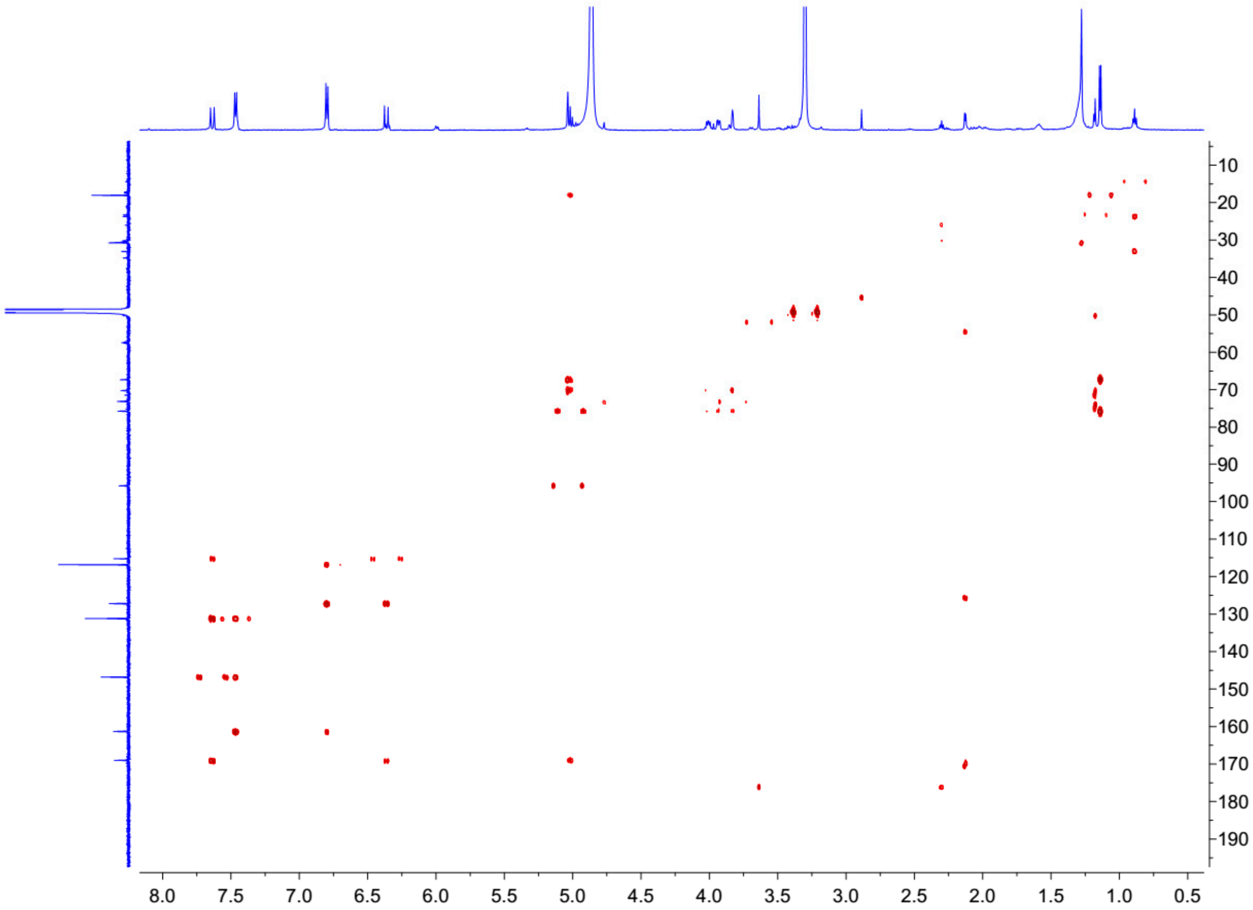

Figure S9. HMBC spectrum and key HMBC correlations of **10**

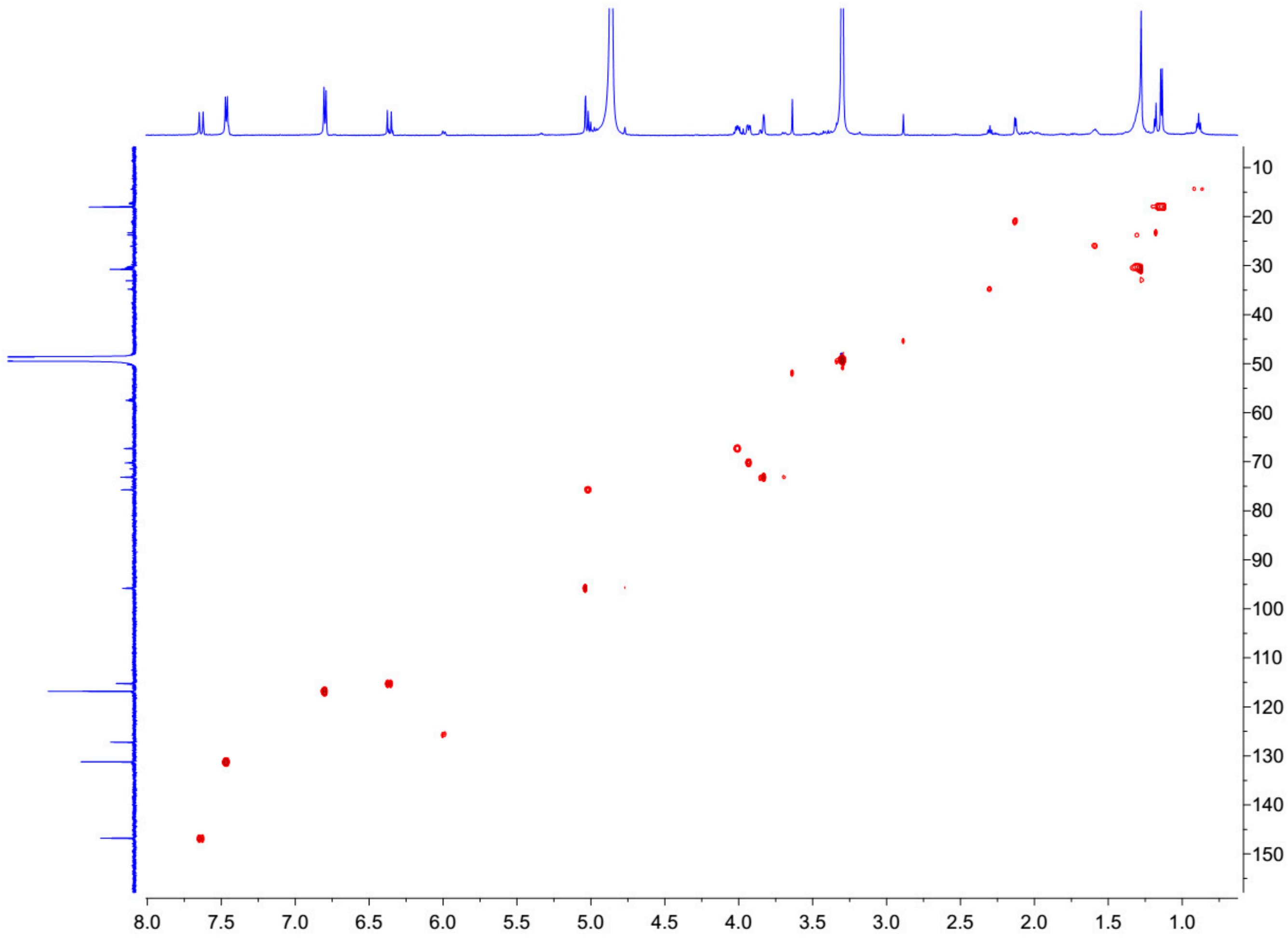

Figure S10. HSQC spectrum of **10**

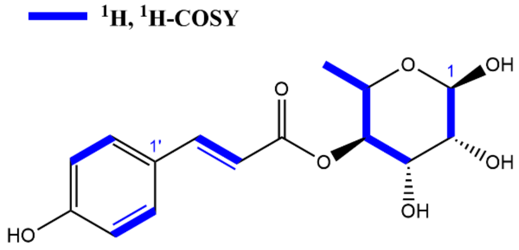

10 4-*O*-(p-coumaryl) rhamnose

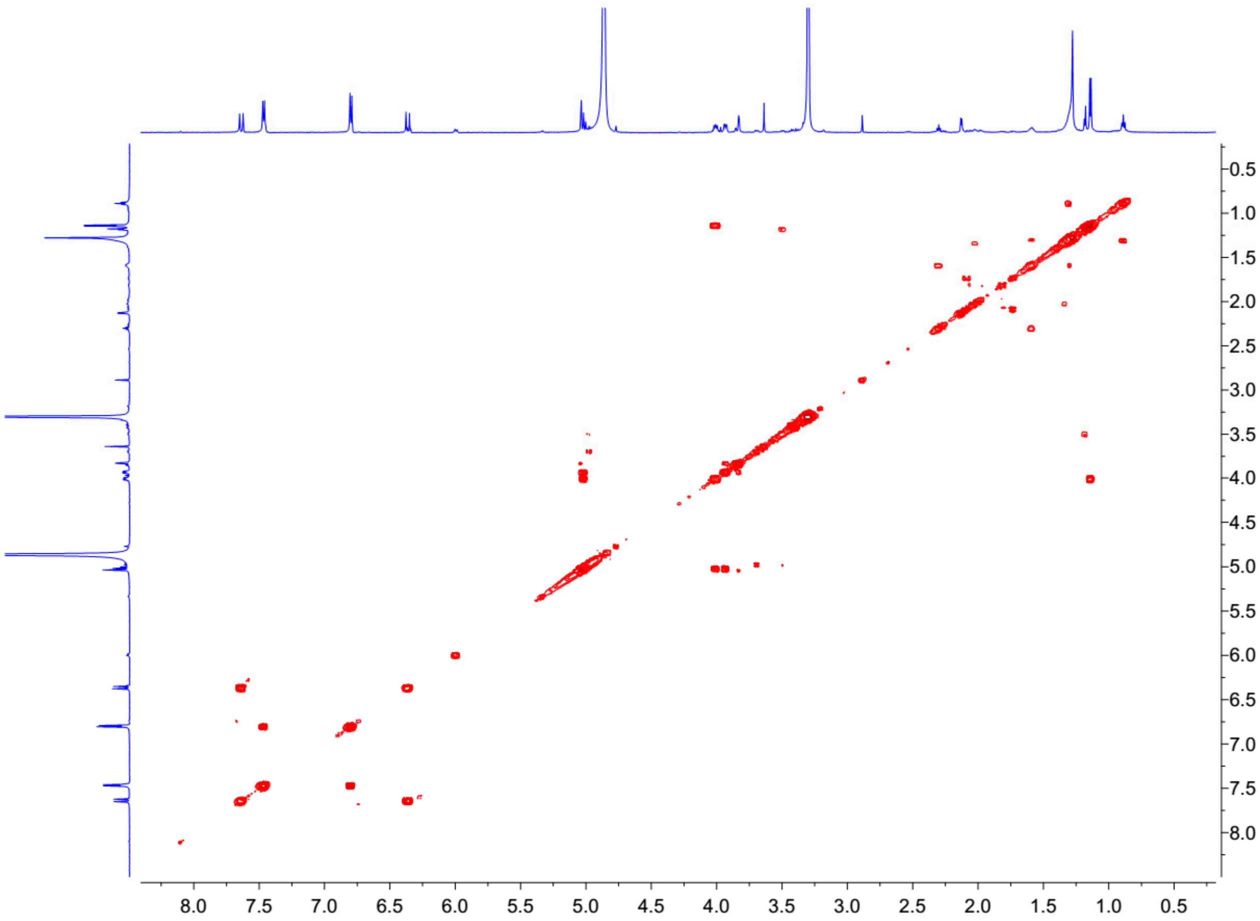

Figure S11. ^{1}H, ^{1}H-COSY spectrum and key ^{1}H, ^{1}H-COSY correlations of **10**

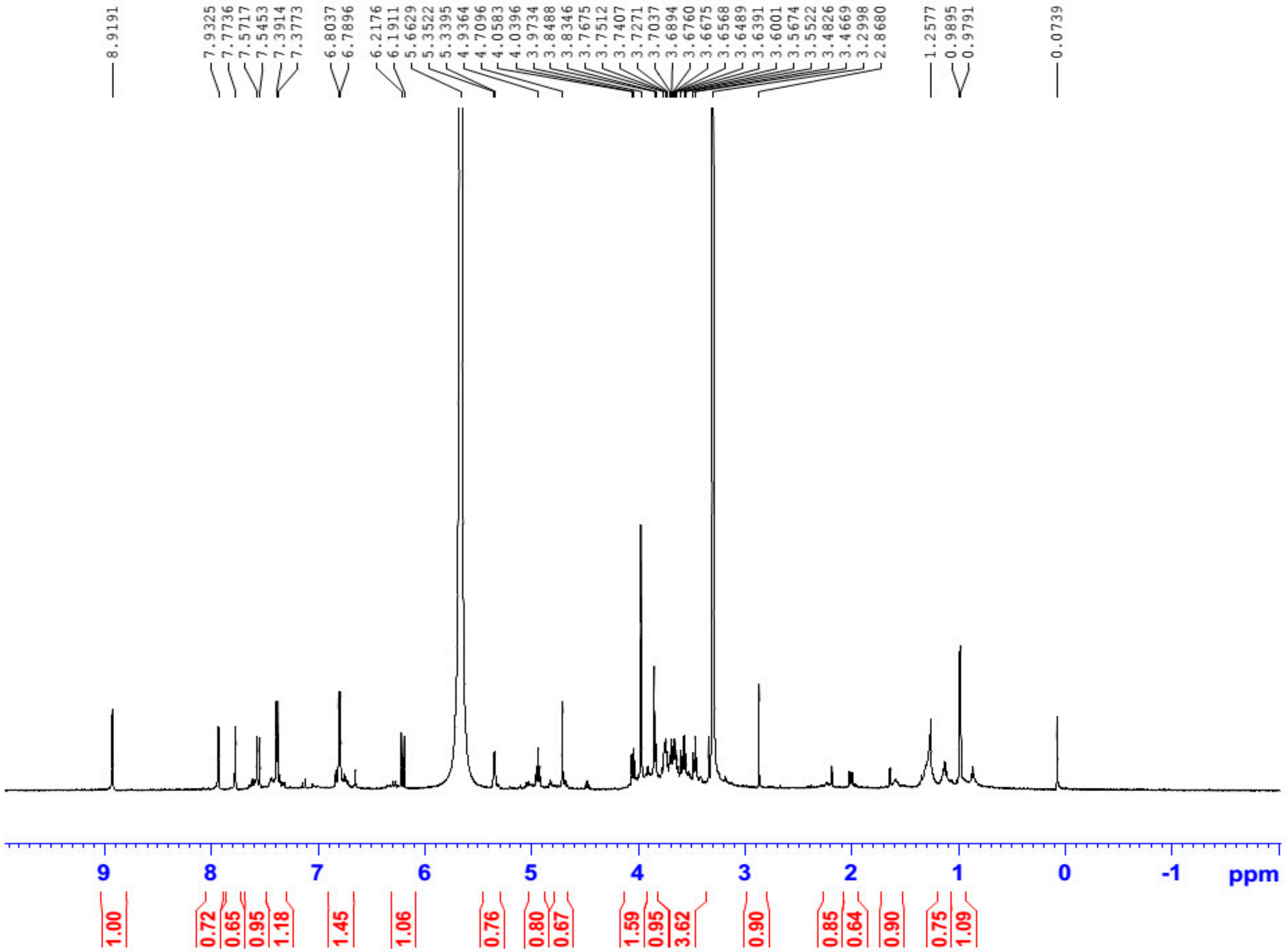

Figure S12. ^{1}H NMR spectrum (600 MHz, CD_3OD/CF_3COOD (9:1)) of **3**

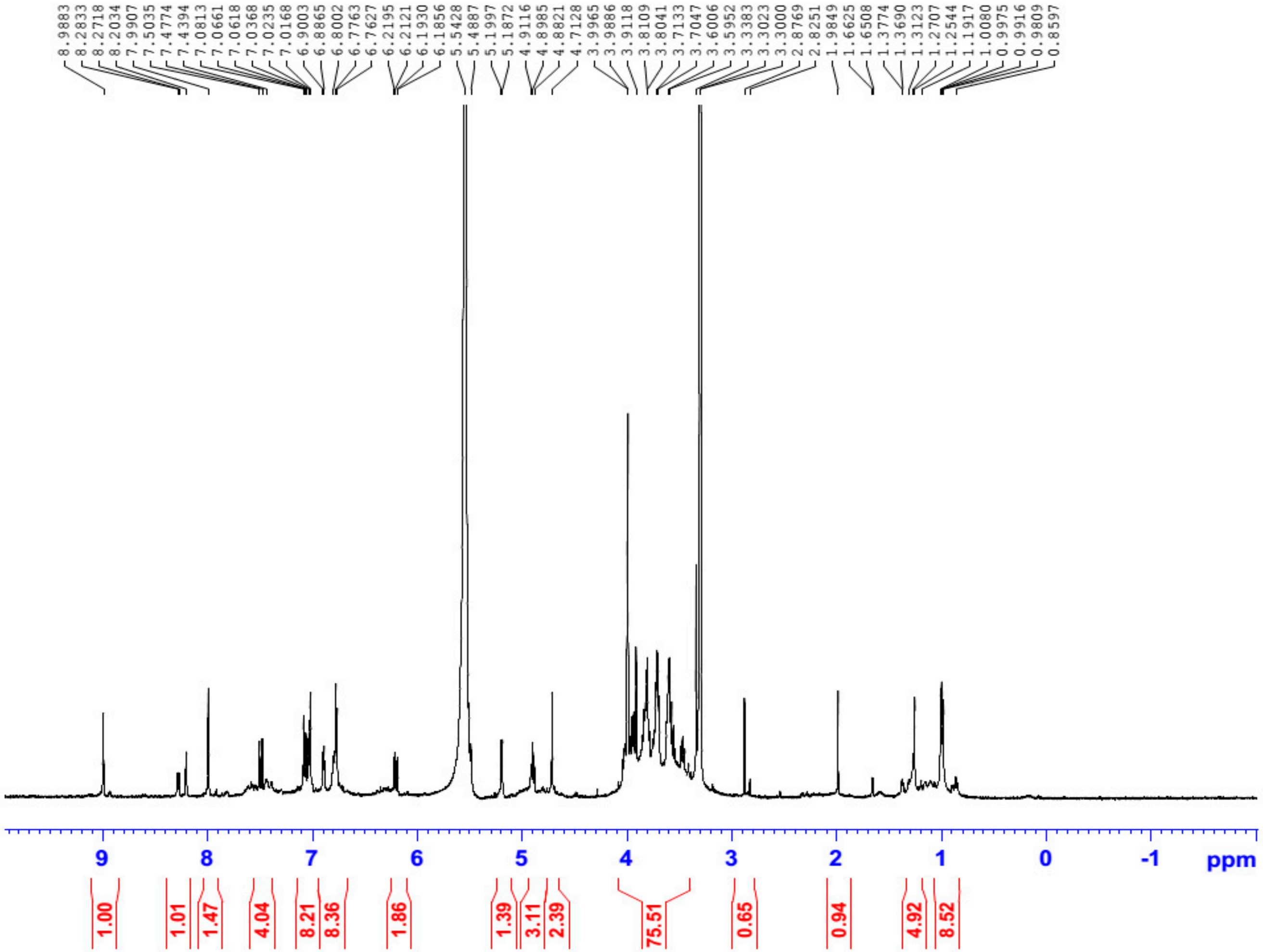

Figure S13. ^{1}H NMR spectrum (600 MHz, CD_3OD/CF_3COOD (9:1)) of **12**

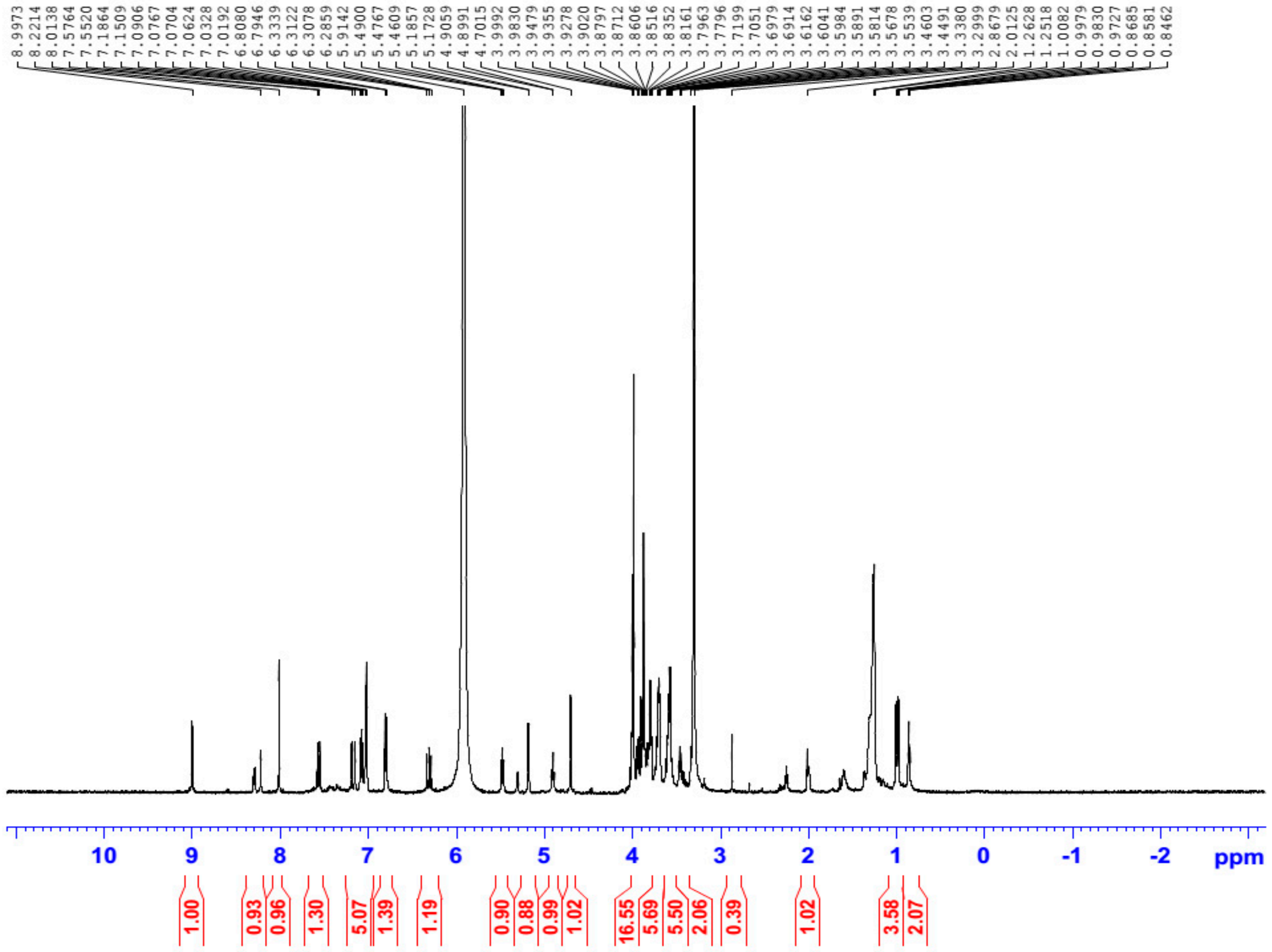

Figure S14. ^{1}H NMR spectrum (600 MHz, CD_3OD/CF_3COOD (9:1)) of **15**

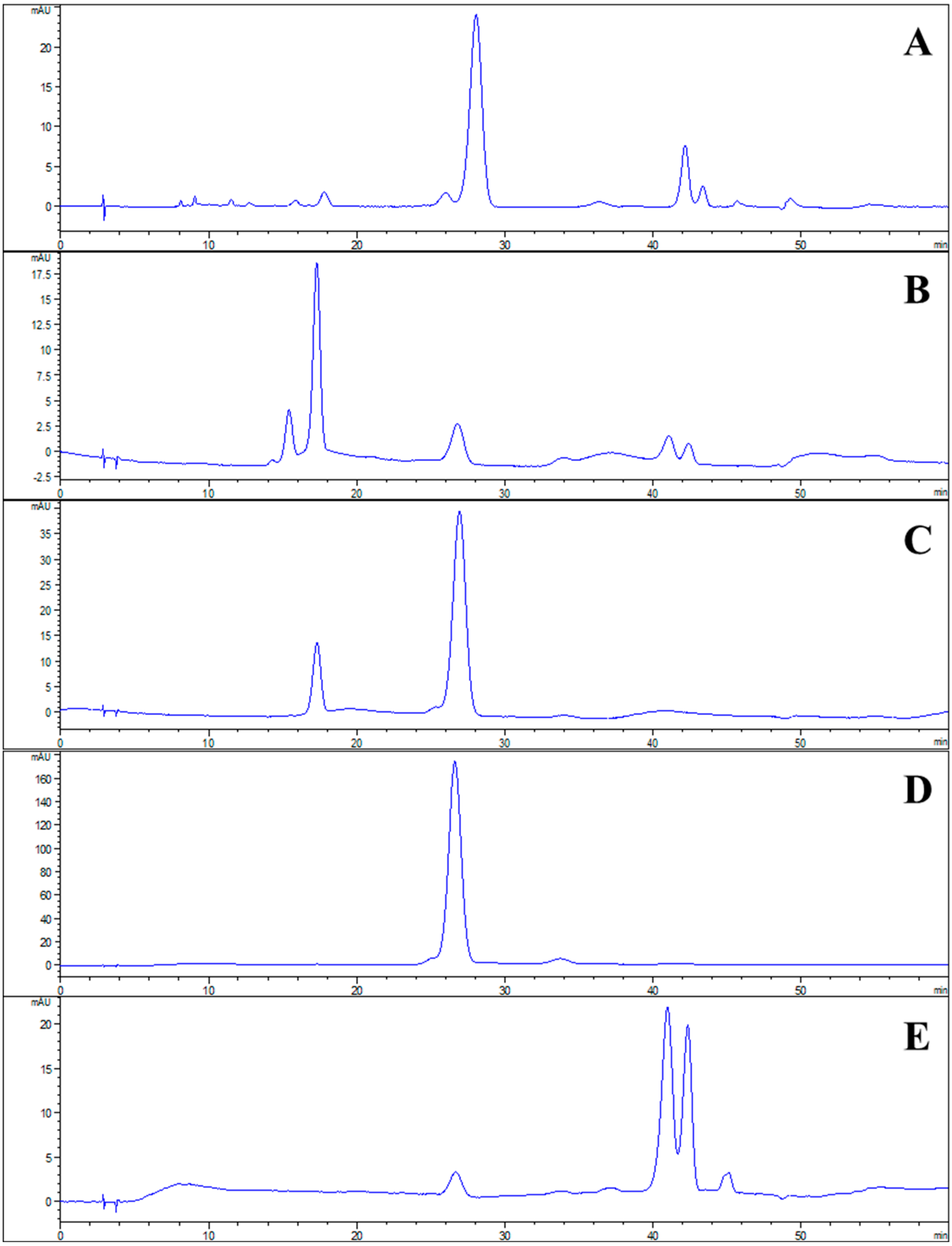

Figure S15. HPLC chromatography of *Solanum tuberosum* L. extract (A) and Fr.1~Fr.4 (B~E)

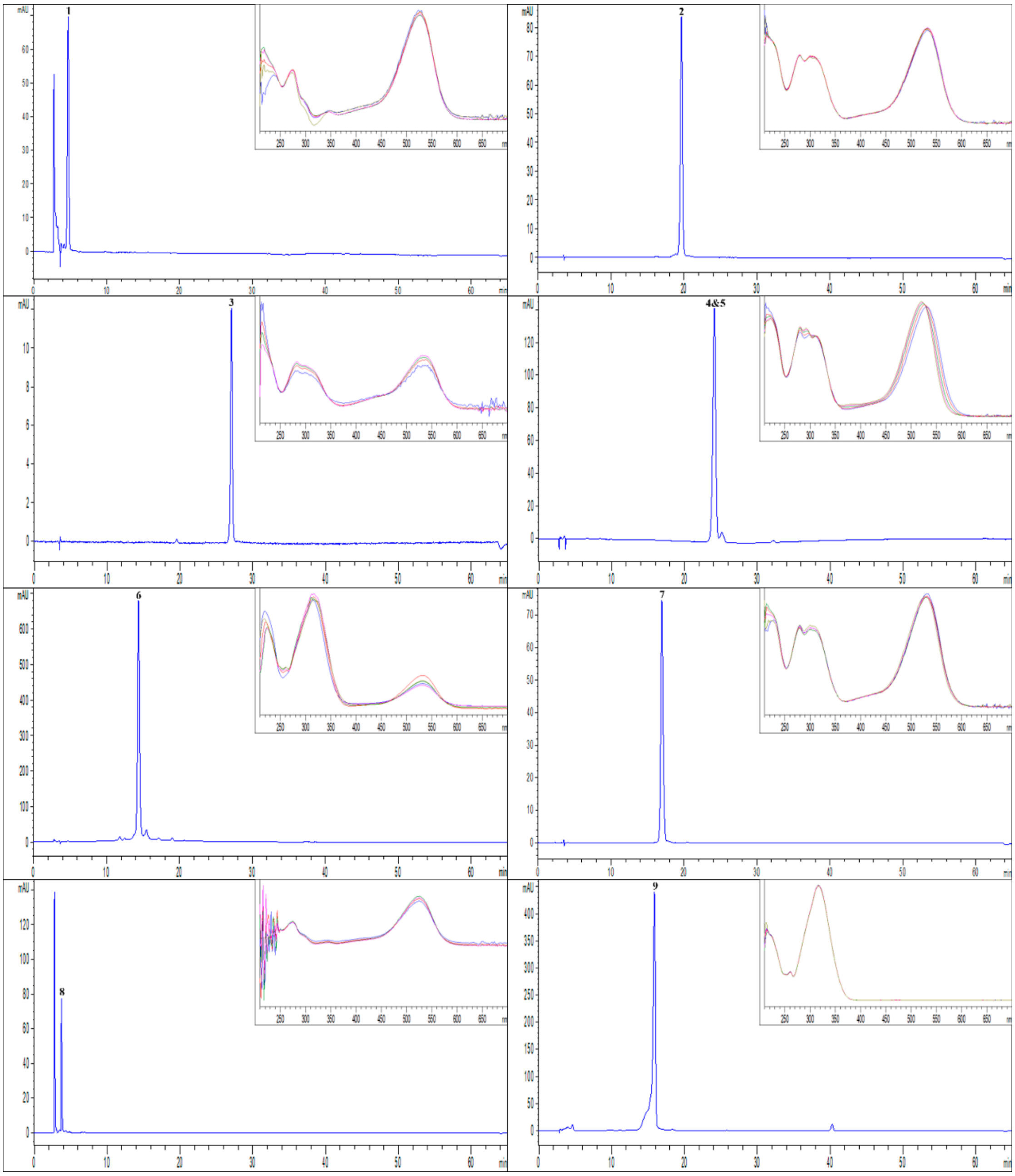

Figure S16. HPLC chromatography and spectrogram of **1~9**

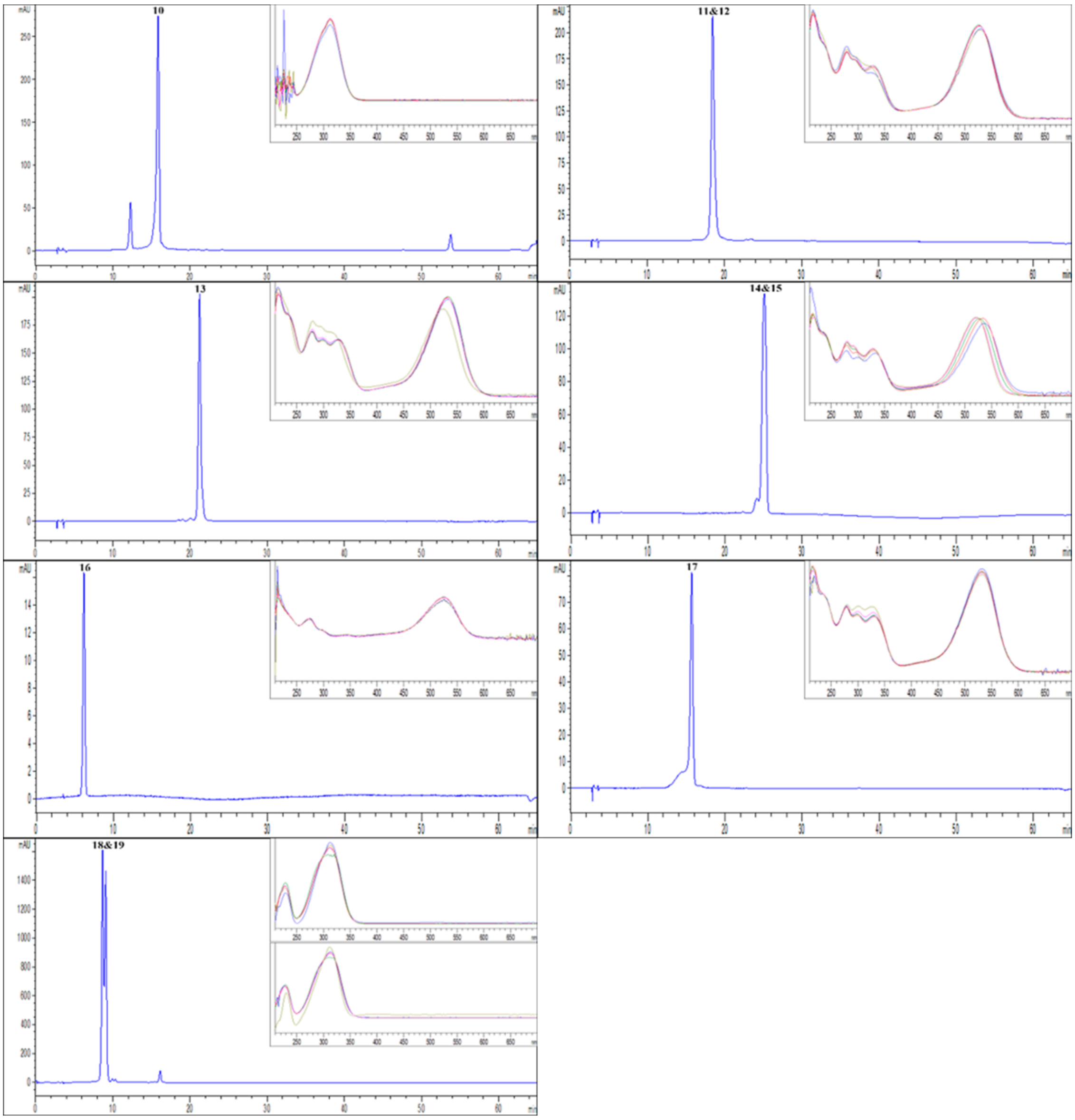

Figure S17. HPLC chromatography and spectrogram of **10~19**

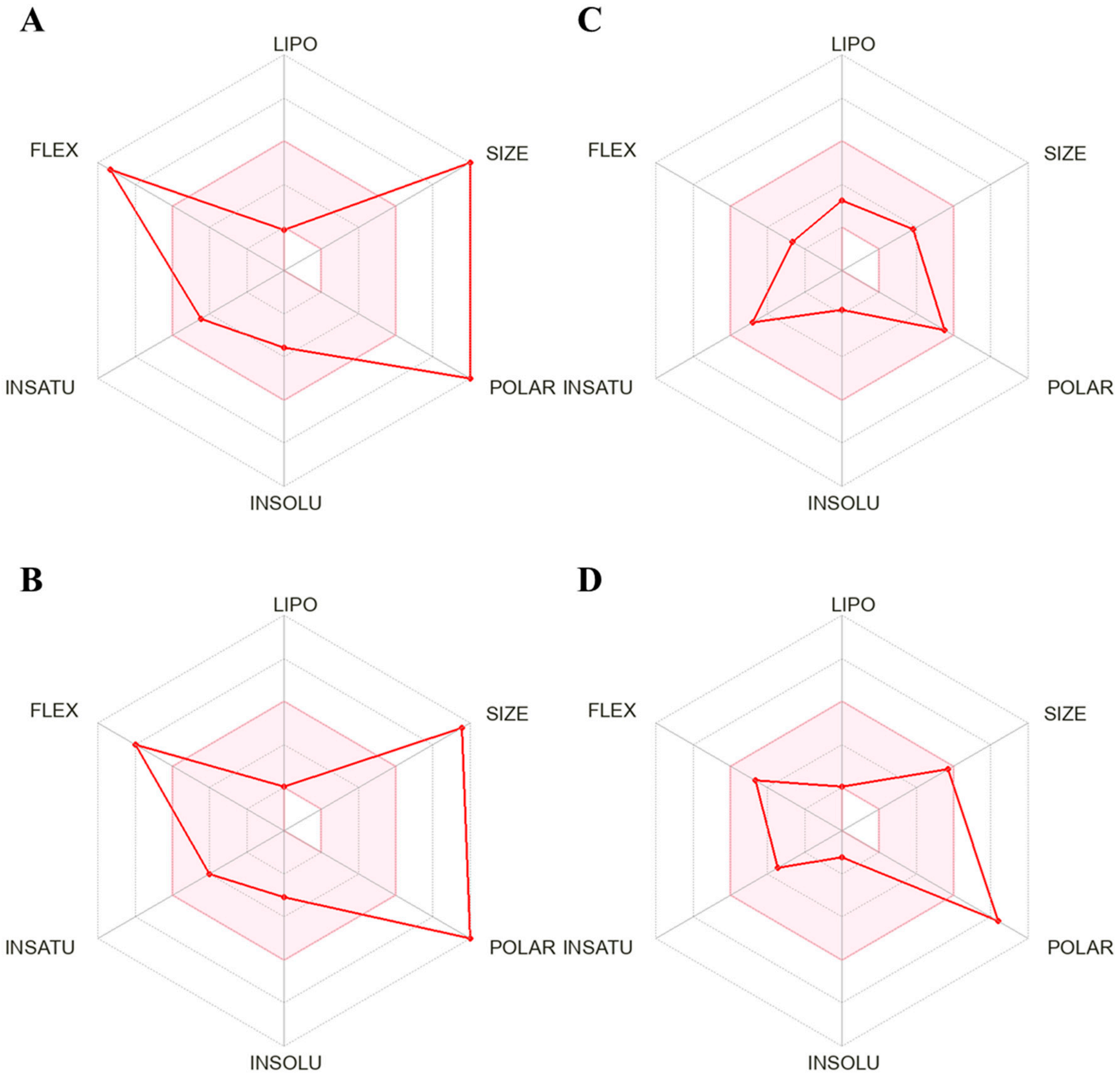

Figure S18. ADMET of (A) petanin, (B) norpetanin, (C) 4-O-(p-coumaryl) rhamnose, (D) Lyciruthephenylpropanoid D/E

Table S1. Retention time, purity and λ_{max} of anthocyanins and their degradation products by HPLC

Compound No.	Retention time (min)	Peak purity (%)	λ_{max} (nm)
**1**	4.731	92.429	272, 528
**2**	19.659	99.433	218, 280, 302, 531
**3**	27.072	100.000	218, 281, 300, 535
**4&5**	24.097	92.654	220, 280, 292, 311, 520
**6**	14.379	91.233	223, 318, 531
**7**	15.564	99.421	228, 280, 300, 310, 530
**8**	3.719	97.602	278, 300, 342, 525
**9**	15.863	94.839	221, 260, 317
**10**	15.854	85.905	313
**11&12**	18.471	97.557	218, 280, 298, 330, 529
**13**	21.241	95.082	218, 280, 300, 330, 530
**14&15**	25.088	94.316	218, 280, 298, 330, 521
**16**	6.169	100.000	273, 300, 341, 528
**17**	15.679	95.536	218, 280, 300, 330, 531
**18&19**	8.671/ 9.082	50.182/ 45.092	230, 311

Table S2. Tyrosinase inhibitory activity of anthocyanins and their degradation products (0.60 mM)

NO.	Compound name	Molecular weight	Inhibitory ratio at 0.60 mM (%)
**1**	Petunidin 3-O-rutinoside-5-O-glucoside	787.2291	83.05 ± 2.76
**2**	**Petanin**	**933.2659**	93.25 ± 0.95
**3**	Petunidin 3-O-trans-p-coumaroylrutinoside	771.2131	64.46 ± 3.09
**4/ 5**	Peonidin 3-O-trans-p-coumaroylrutinoside-5-O-glucoside<br>Malvidin 3-O-trans-p-coumaroylrutinoside-5-O-glucoside	917.2710<br>947.2816	66.74 ± 2.79
**6**	Petunidin 3-O-trans-caffeoylrutinoside-5-O-glucoside	949.2608	87.01 ± 2.59
**7**	Delphinidin 3-O-trans-p-coumaroylrutinoside-5-O-glucoside	919.2503	79.79 ± 2.35
**8**	Delphinidin 3-O-rutinoside-5-O-glucoside	773.2135	86.47 ± 1.75
**9**	**Norpetanin**	**810.2219**	94.31 ± 2.36
**10**	**4-O-(p-coumaryl) rhamnose**	**310.1053**	73.56 ± 0.90
**11/ 12**	Malvidin 3-O-trans-caffeoylrutinoside-5-O-glucoside<br>Peonidin 3-O-trans-caffeoylrutinoside-5-O-glucoside	963.2765<br>933.2659	47.01 ± 3.19
**13**	Petunidin 3-O-feruloylrutinoside-5-O-glucoside	963.2765	48.83 ± 2.59
**14/ 15**	Malvidin 3-O-feruloylrutinoside-5-O-glucoside<br>Peonidin 3-O-feruloylrutinoside-5-O-glucoside	977.2921<br>947.2816	67.29 ± 2.85
**16**	Petunidin 3,5-di-O-glucoside	641.1712	90.40 ± 1.58
**17**	Peonidin 3-O-rutinoside-5-O-glucoside	771.2342	57.79 ± 2.81
**18&19**	**Lyciruthephenylpropanoid D**<br>**Lyciruthephenylpropanoid E**	**472.1581**	90.30 ± 4.79

Table S3. Maximum tolerated concentration of Petanin in zebrafish (n=30)

**Group**	**Content （%）**	**Death count (Tail)**	**Death rate (%)**	**Phenotype**
**Normal**	/	0	0	No obvious abnormality
**Petanin**	0.002	0	0	ns
	0.02	0	0	ns
	0.05	0	0	ns
	0.1	0	0	ns
	**0.15**	**1**	**3**	**ns**
	0.20	6	20	/
	0.25	12	40	/
	0.5	30	100	/

Note: ns means that there is no significant difference compared with the normal group.
